# Insulin-like growth factor receptor and sphingosine kinase are prognostic and therapeutic targets in breast cancer

**DOI:** 10.1186/s12885-017-3809-0

**Published:** 2017-12-05

**Authors:** Aleksandra M. Ochnik, Robert C. Baxter

**Affiliations:** 1 Kolling Institute, University of Sydney, Royal North Shore Hospital, St Leonards, NSW 2065 Australia; 2 Centre for Drug Discovery & Development, Sansom Institute for Health Research, School of Pharmacy & Medical Sciences, University of South Australia, Adelaide, South Australia 5001 Australia

**Keywords:** Insulin-like growth factor receptor, Breast cancer, Targeted-therapies and sphingosine kinase

## Abstract

**Background:**

Targeting the type 1 insulin-like growth factor receptor (IGF1R) in breast cancer remains an ongoing clinical challenge. Oncogenic IGF1R-signaling occurs via activation of PI3K/AKT/MAPK downstream mediators which regulate cell proliferation and protein synthesis. To further understand IGF1R signaling we have investigated the involvement of the oncogenic IGF1R-related sphingosine kinase (SphK) pathway.

**Methods:**

The prognostic (overall survival, OS) and therapeutic (anti-endocrine therapy) co-contribution of IGF1R and SphK1 were investigated using breast cancer patient samples (*n* = 236) for immunohistochemistry to measure total and phosphorylated IGF1R and SphK1. Kaplan-Meier and correlation analyses were performed to determine the contribution of high versus low IGF1R and/or SphK1 expression to OS in patients treated with anti-endocrine therapy. Cell viability and colony formation in vitro studies were completed using estrogen receptor (ER) positive and negative breast cancer cell-lines to determine the benefit of IGF1R inhibitor (OSI-906) and SphK inhibitor (SKI-II) co-therapy. Repeated measures and 1-way ANOVA were performed to compare drug treatments groups and the Chou-Talalay combination index (CI) was calculated to estimate drug synergism in vitro (CI < 1).

**Results:**

High IGF1R and SphK1 protein co-expression in tumor tissue was associated with improved OS specifically in ER-positive disease and stratified for anti-endocrine therapy. A significant synergistic inhibition of cell viability and/or colony formation following OSI-906 and SKI-II co-treatment in vitro was evident (*p* < 0.05, CI < 1).

**Conclusion:**

We conclude that high IGF1R and SphK1 co-expression act together as prognostic indicators and are potentially, dual therapeutic targets for the development of a more effective IGF1R-directed combination breast cancer therapy.

**Electronic supplementary material:**

The online version of this article (10.1186/s12885-017-3809-0) contains supplementary material, which is available to authorized users.

## Background

Clinically targeting oncogenic signaling pathways in breast cancer, such as those initiated by the estrogen receptor (ER) and the human epidermal growth factor receptor-2 (HER2), has been highly beneficial to the treatment of the disease. However, given the heterogeneity that exists among breast cancer molecular subtypes based on the ER, progesterone receptor (PR) and HER2 status, which modulate many growth factor signaling pathways such as type 1 insulin-like growth factor receptor (IGF1R) signaling [1, 2], it is evident that singularized breast cancer targeted therapies are associated with therapeutic drawbacks such as a propensity to develop therapy resistance [3–5].

Specifically, the IGF1R signaling pathway has been shown to play an oncogenic role in both ER-positive and ER-negative breast cancer via the activation of downstream PI3K/AKT/MAPK/FAK signaling mediators to effectively regulate cell proliferation, migration and protein synthesis (i.e. mRNA translation) [6, 7]. However, in conflict with the oncogenic role of IGF1R signaling, lowe IGF1R expression has been reported to be associated with poorer outcomes in ER-negative breast cancer [8], compared to high IGF1R expression which leads to a better outcome [8, 9]. Moreover, high phosphorylated IGF1R (p-IGF1R) expression in luminal, triple-negative, and HER2 subtypes combined has been shown to be associated with a poorer survival outcome suggesting that IGF1R activation compared to expression may be more important as a prognostic factor [10]. Despite the pre-clinical evidence suggesting that therapeutically targeting the IGF1R-pathway would be clinically effective in some patients, IGF1R monotherapies to date have not shown any improvements in clinical outcome and there is still a need to identify specific IGF1R co-related prognostic factors and therapeutic approaches [6, 11, 12]. Moreover, there is still conflicting prognostic vs. preclinical data in relation to the benefits of IGF1R targeted therapies in breast cancer which highlights the need for a better understanding of IGF1R signaling [12].

In addition to the ER-signaling pathway, IGF1R is known to regulate the oncogenic lipid kinase, sphingosine kinase 1 (SphK1) pathway which mediates proliferative, migratory and angiogenic effects. These effects are mediated via the intracellular and extracellular actions of the second messenger prosurvival lipid sphingosine 1-phosphate (SIP) and the SIP receptors, S1P1-S1P5 located in the plasma membrane in breast cancer [13–18]. SphK1 is known to be expressed in both ER positive and negative breast cancer and is associated with worse disease outcomes in both [19, 20].

Pre-clinical studies using SphK1-targeting therapies have shown that they possess anticancer activity, and recent findings have demonstrated that co-treatment with an epidermal growth factor receptor (EGFR) targeted therapy, gefitinib, and SphK1-targeted therapy has clinical potential in breast cancer [21–24]. Moreover, expression of IGF1R and SphK1/SIP-receptors has been shown to contribute to tamoxifen resistance in ER-positive breast cancer [16, 25, 26] which further highlights the need to better understand the significance of IGF1R and SphK1 co-expression and their contribution to anti-estrogen therapy resistance in breast cancer.

In order to further understand the prognostic and therapeutic implications of IGF1R and SphK1 co-expression in breast cancer we have analyzed their distribution in human breast cancer formalin-fixed paraffin embedded (FFPE) tissue samples. In addition we have undertaken pre-clinical in vitro studies using the dual IGF1R/insulin receptor (InsR) tyrosine kinase inhibitor, OSI-906 and the SphK inhibitor, SKI-II as a novel IGF1R-directed combination therapy. This study has identified novel relationships between breast cancer patient survival outcome and ER, PR and HER2 status and anti-estrogen therapy, based on IGF1R and SphK1 protein expression. Moreover, our evidence in vitro suggests that therapeutically co-targeting IGF1R and SphK1 has the potential for clinical benefit. In line with the findings of this study, IGF1R and SphK1 expression may have prognostic significance and co-directed combination therapies may be beneficial, specifically for ER-positive breast cancer.

## Methods

### Reagents and drugs

Cell culture reagents were purchased from Trace Biosciences (North Ryde, New South Wales, Australia) and Nunc (Roskilde, Denmark). Bovine insulin, methanol, calcium chloride, magnesium chloride, crystal violet powder and 1-(4,5-Dimethylthiazol-2-yl)-3,5-diphenylformazan were purchased from Sigma-Aldrich. Enhanced chemiluminescence (ECL) reagent was SuperSignal West Pico Chemiluminescent Substrate (Pierce Biotechnology). The dual IGF1R/insulin receptor tyrosine kinase inhibitor (OSI-906; also referred to as linsitinib) was purchased from MedChem Express (Princeton, NJ) and the SphK inhibitor 2-(p-hydroxyanilino)-4-(p-chlorophenyl)thiazole (SKI-II) from Calbiochem [21]. Antibodies raised against phospho-Y1135/1136 IGFR1, IGFR1 beta chain, phospho-Ser473 AKT and total AKT, 4E-BP1 and eIF4E were purchased from Cell Signaling Technology (Beverley, MA). The antibody to detect SphK1 (ab16491) for western blots was purchased from Abcam and for SphK1 immunohistochemistry, from Abgent (AP7237c).

### Patient cohort

Breast cancer tissues were obtained from the Australian Breast Cancer Tissue Bank (ABCTB), Westmead, NSW, Australia for the purposes of this study. This study was approved by the Human Research Ethics Committee of the Northern Sydney Local Health District (Reference Numbers: RESP/15/125 and LNR/15/HAWKE/182) for the analysis of human breast cancer tissues samples obtained from the Australian Breast Cancer Tissue Bank. All samples obtained from this bank were de-identified and were from donors who had given written informed consent for their banked tumor tissue to be used in future research projects.

A total of 236 FFPE breast tissue samples were approved for use, comprised of five tissue micro-arrays (TMA) in duplicate or triplicate cores (0.6 mm^3^) (187 patients in total) and 49 whole face tissue sections. All patient samples had molecular subtyping from the ABCTB for ER, PR and HER2 expression by IHC and/or FISH analysis (for HER2) (Table 1). Patient information provided by the ABCTB included gender, disease status (all reported as invasive), pathology notes where applicable, primary histologic diagnosis and histopathological grade (Table 1). Patient follow-up data provided by the ABCTB consisted of diagnosis age, year of first breast event, time of follow-up since diagnosis and follow-up status (median follow up; 61 months (Table 1). In addition the ABCTB provided information relating to the therapy the patients received included the following: 1) anti-endocrine therapy; 2) HER2-therapy and 3) chemotherapy (Table 2). All studies were performed with approval from the Northern Sydney Local Health District (NSLHD) Human Research Ethics Committee (HREC), which assessed it as a low-negligible risk study.

**Table 1:** Patient Clinicopathologic Characteristics (*n* = 236)

Gender	*n* (%)
Female	233 (98.7)
Male >51y	3 (1.3)
Age (Female)	
< 51y	93 (40.0)
≥ 51y	140 (60.0)
Age	
20-29	4 (1.7)
30-39	27 (11.4)
40-49	55 (23.3)
50-59	68 (28.8)
60-69	49 (20.8)
70-79	25 (10.6)
80-89	8 (3.4)
Histopathology	
IDC	197 (83.5)
ILC	16 (6.8)
Apocrine Carcinoma	5 (2.1)
Medullary Carcinoma	3 (1.3)
Mucinous Carcinoma	3 (1.3)
Basal-like Carcinoma	2 (0.8)
Tubular Cancer	2 (0.8)
ILC/Tubulolobular	1 (0.4)
Tubulolobular	1 (0.4)
Mixed Carcinoma	1 (0.4)
Papillary Carcinoma	1 (0.4)
Infiltrating	1 (0.4)
Other	3 (1.3)
Grade	
Invasive grade I	22 (9.3)
Invasive grade II	65 (27.5)
Invasive grade III	149 (63.1)
Molecular Subtype	
ER+, PR+, HER2+	41 (17.4)
ER+, PR+, HER2-	95 (40.3)
ER+, PR-, HER2-	11 (4.7)
ER+, PR-, HER2+	12 (5.1)
ER-, PR+, HER+	6 2.5)
ER-, PR+, HER2-	2 (0.8)
ER-, PR-, HER2+	24 (10.2)
ER-, PR-, HER2-	35 (14.8)
Equivocal/Not performed	10 (4.2)
Equivocal/Not performed: Lack of result for ER, PR and/or HER2)	
Disease Outcome/Follow-Up Status	n (%)
Died From Disease	13 (5.5)
Died From Other Causes	8 (3.4)
Overall Died	21 (8.9)
Alive With Disease	6 (2.5)
Alive With No Disease	207 (87.7)
Alive Disease Status Unknown	2 (0.8)
Overall Alive	215 (91.1)

**Table 2:** Patient Therapy (*n* = 236)

Therapy	*n* (%)
Anti-Endocrine Therapy = 165 (69.9)	
Tamoxifen	75 (31.7)
Anastrozole (Arimidex)	45 (19)
Exemestane (Aromasin)	8 (3.3)
Letrozole (Femara)	32 (13.5)
Goserelin (Zoladex)	4 (1.6)
Aromatase Inhibitors	1 (0.4)
HER2 Therapy = 67 (28.4)	
Trastuzumab (Herceptin)	66 (27.9)
Lapatinib (Tyverb)	1 (0.4)
Chemotherapy	194 (82.2)
AC: Adriamycin (Doxorubicin), Cyclophosphamide	49 (20.7)
Anthracycline	1 (0.4)
Docetaxel (Taxotere)	19 (8.0)
TAC: Docetaxel (Taxotere), Adriamycin (Doxorubicin) and Cyclophosphamide	22 (9.3)
TC: Docetaxel (Taxotere) and Cyclophosphamide	1 (0.4)
TCH: Docetaxel (Taxotere), Carboplatin and Trastuzumab	9 (3.8)
EC: Epirubicin and Cyclophosphamide	3 (1.2)
FEC: 5-Fluorouracil, Epirubicin and Cyclophosphamide	45 (19.0)
Paclitaxel (Taxol)/Taxane	36 (15.2)
FAC (or CAF): 5-Fluorouracil, Doxorubicin and Cyclophosphamide	4 (1.6)
Capecitabine (Xeloda)	3 (1.2)
5-Fluorouracil	1 (0.4)
Carboplatin	1 (0.4)
Unknown	46 (19.4)

### Immunohistochemistry

IHC was performed on 4 μm FFPE sections using an automated tissue stainer (Autostainer, DAKO, Glostrup, Denmark) according to standard manufacturer’s operating procedures. Antigen retrieval was performed using a water bath heated to 99.2 °C for 20 min in freshly made 10 mM citric acid monohydrate adjusted to pH 6.0. The sections were quenched in 0.3% hydrogen peroxide for 5 min, blocked with 5% goat serum for 30 min and incubated in primary antibodies: IGF1Rβ antibody (no cross-reaction with the insulin receptor (InsR)) (#3027, Cell Signaling, Danvers, MA, USA 1:100), p-IGF1R (#ab39398, Abcam, Melbourne, VIC, Australia, 1:200) and SphK1 (#AP7237c, Abgent, San Diego, CA, USA, 1:200) for one hour at room temperature. Protein detection was subsequently performed using the DAKO-Envision Dual Link Labelled Polymer (Anti-Rabbit) (#K5007, Dako, Botany, NSW, Australia) for 30 min and the ImmPACT NovaRed Peroxidase Substrate Kit (#SK-4805, Vector Laboratories, Burlingame, CA, USA) for 10 min at room temperature. All antibodies were optimized using a series of dilutions on a TMA comprised of ten ER-positive and negative breast cancer patient tissues in duplicate to determine an optimal dilution for IHC staining. Final dilutions were closely assessed for specific membranous, cytoplasmic and/or nuclear staining in line with the literature. Negative controls were included for all IHC using a rabbit immunoglobulin fraction (#X0936, DAKO) at the final concentrations of the primary antibodies and a tissue sample incubated with the anti-rabbit antibody in the absence of a primary antibody.

### Manual scoring

**Fig. 1:**
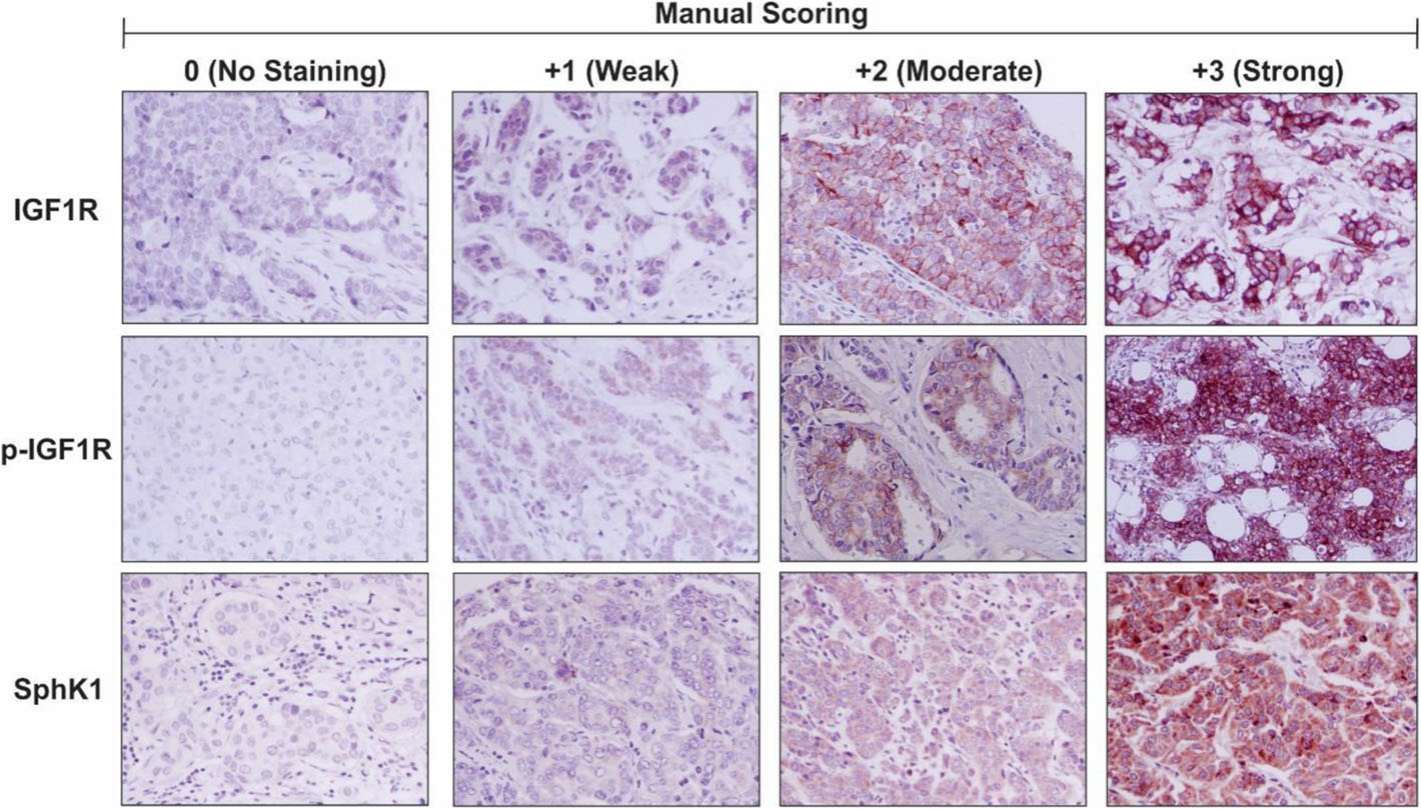
Immunohistochemistry and manual scoring analysis of Australian Breast Cancer Tissue Bank patient samples. Immunohistochemistry was performed on formalin-fixed paraffin embedded breast cancer patient tissue samples (*n* = 236) obtained from the Australian Breast Cancer Tissue Bank (ABCTB) using antibodies to detect and measure relative levels of IGF1R, p-IGF1R and SphK1. The intensity of immunostaining was assessed by manual scoring according to standard guidelines as follows: 0 = no staining, 1 = weak staining, 2 = moderate staining and 3 = strong staining for IGF1R, p-IGF1R and SphK1

Manual scoring was assessed on all samples for subsequent statistical analysis, with examples shown in Fig. 1. IGF1R and p-IGF1R staining expression levels were manually assessed using the HER2 scoring system described in the Hercep Test manual (DAKO) as follows: no staining = 0, faint staining = 1, weak to moderate staining = 2 and strong staining = 3, in line with published studies [27, 28]. Positive staining was defined as membrane/cytoplasmic and/or nuclear staining detectable in ≥10% of tumor epithelial cells. SphK1 staining levels were manually assessed as: no staining (<10% of tumor epithelial cells with cytoplasmic staining) = 0, weak = 1, moderate = 2 and strong = 3, in accordance with previously published data [29–31].

### Survival analysis

Kaplan-Meier (KM) survival analysis was performed using SPSS v.22 (IBM, Armonk, NY, USA) to determine prognostic significance of high protein expression of phospho- and total IGF1R, and SphK1, for overall survival (OS) and disease-free survival (DFS) in non-stratified patient cohorts, and after stratification for high or low ER, PR, and HER2. KM analysis was also performed to determine the relationship between IGF1R and SphK1 protein expression in patients stratified for anti-endocrine therapy treatment. For KM analysis of IGF1R, p-IGF1R, and SphK1, staining scores described above, on a scale of 0 to 3, were converted to a new binary scale where 0 or 1 = low, and 2 or 3 = high. For analysis of IGF1R-SphK1 and p-IGF1R-SphK1 co-expression, a binary co-expression score was used: either or both analytes low = low co-expression, both analytes high = high co-expression. Significance (*P* < 0.05) was determined by the log-rank test. Multivariate survival analysis was performed in SPSS by the Cox proportional hazards method, using forward stepwise (likelihood ratio) regression, with significance set at *P* < 0.05. To determine the relationship between IGF1R and SphK1 in non-stratified and stratified patient groups including: ER+/−, PR+/− and HER2+/−, age, tumor grade correlation analysis was performed in SPSS using Spearman correlation analysis with statistical significance set at P < 0.05. All data used in these analyses are included as Additional file 1: Table S1.

### Breast cancer cell-lines and culture

The ER-positive MCF7 and T47D and ER-negative HCC1806 and HCC70 breast cancer cell-lines were purchased from the American Tissue Culture Collection (Manassas, VA, USA) and cultured and maintained in phenol-red RPMI-1640 medium supplemented with 5% FBS, 15 mM Hepes and 10 mg/mL bovine insulin at 37 °C in humidified 5% CO_2_ atmosphere.

### MTT-assay and Clonogenic survival assay

The MTT (3-(4,5-dimethylthiazol-2-yl)-2,5-diphenyltetrazolium bromide) assay and clonogenic survival assay were performed by plating 2 × 10^3^ MCF7, T47D, HCC1806 and HCC70 cells per well of a 96-well plate (MTT-assay) or a 6-well plate (clonogenic assay) in phenol-red RPMI culture media supplemented with 5% FBS and culturing for 24 h. The cells were treated with culture media containing 5% FBS in addition to OSI-906 (0, 0.1, 0.4, 1.6 or 6.4 μM) and/or SKI-II (0, 0.16, 0.8, 4, 10 or 20 μM) in combination for 96 h for MTT-assay and 10-14 days for clonogenic assay. For the MTT-assay 30 μl of 1-(4,5-dimethylthiazol-2-yl)-3,5-diphenylformazan (thiazolyl blue formazan) solution prepared at 2.5 mg/ml, in PBS (containing 0.9 mM calcium chloride plus 0.5 mM magnesium chloride) was added per well and incubated at 37 °C for 4 h. The cells were solubilized in 150 μl of DMSO for 15 min at room temperature (RT). Absorbance was read using a plate reader at 460 nm. For the clonogenic assay, the cells were washed twice in PBS (0.9 mM calcium chloride plus 0.5 mM magnesium chloride) solution at RT, fixed in 0.1% crystal violet prepared in final 20% methanol solution and destained in tap water. The cells were left to air-dry overnight and images obtained using FujiFilm Luminescent Image Analyzer LAS-300 and single colonies were counted using open colony forming unit (CFU) software (http://opencfu.sourceforge.net/).

### Immunoblot

3 × 10^5^ MCF7 and HCC-1806 breast cancer cell-lines were plated per well of 6-well plates, cultured for 24 h and subsequently treated with OSI-906 (0.1, 0.4 or 1.6 μM) and/or SKI-II (4 μM) for 24 h. Protein lysates were prepared and 20 μg of protein was further run on SDS-polyacrylamide gels, transferred to Hybond C nitrocellulose and probed with antibodies to detect phospho and/or IGF1R, AKT, 4E-BP1, eIF4E (Cell Signaling) and SphK1 (Abgent) steady-state protein levels as previously described [32]. Β-actin antibody was used as a loading control. Subsequent to the addition of the ECL-reagent, a FujiFilm Luminescent Image Analyzer LAS-300 (Stamford, CT) was used for band detection and image production.

### Statistical analysis (cell culture)

The effect of drug treatments on MTT and clonogenic assays was first analysed across the full OSI-906 dose-range using 2-way ANOVA for repeated measures (SPSS) with experiment and SKI-II dose as factors, and OSI-906 dose as the repeated measure. Comparison of individual dose combinations was performed using 1-way ANOVA with Tukey’s post-hoc test. (GraphPad Prism v.7, La Jolla, CA, USA). Statistical significance was defined as a *p*-value <0.05*, *p* < 0.01**, *p* < 0.001*** and *p* < 0.0001****. Drug synergism was determined using the Chou Talalay method to calculate the combination index (CI) <1 [33].

## Results

### IGF1R expression is positively associated with overall survival in breast cancer

**Fig. 2:**
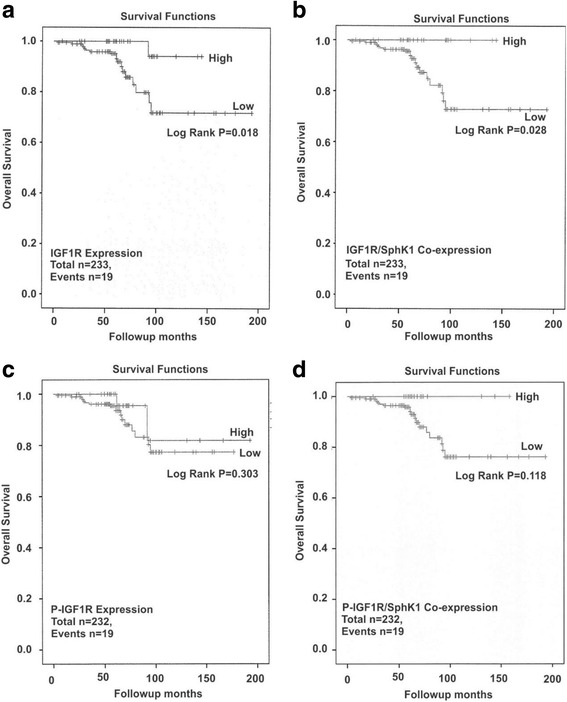
Survival outcomes in relation to p-IGF1R, IGF1R and/or SphK1 protein expression in breast cancer patients. Kaplan-Meier analysis was performed to measure the overall survival (OS) following stratification for high vs. low p-IGF1R, IGF1R and SphK1 protein expression as follows: **a**. IGF1R; **b**. p-IGF1R; **c**. IGF1R and SphK1 co-expression and **d**. p-IGF1R and SphK1 co-expression as described under Methods

**Fig. 3:**
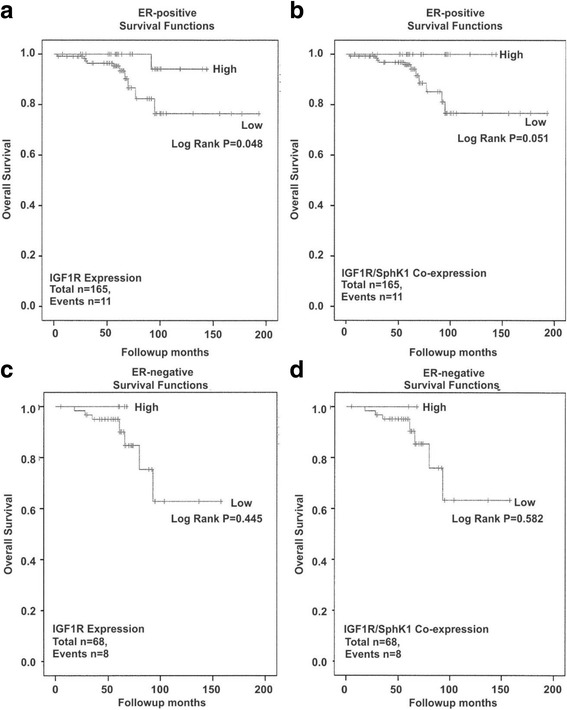
Survival outcomes in relation to p-IGF1R, IGF1R and/or SphK1 protein expression in ER positive and negative breast cancer tissues. Kaplan-Meier analysis was performed to measure the overall survival (OS) following stratification for high vs. low IGF1R, SphK1 protein expression stratified for ER expression as follows: **a**. IGF1R (ER-positive); **b**. IGF1R and SphK1 co-expression (ER-positive); C. IGF1R (ER-negative) and D. IGF1R and SphK1 (ER-negative) as described under Methods

In FFPE tissue sections, high total IGF1R protein expression, in isolation or combined with SphK1, was associated with a better overall survival (OS) rate when analyzed across all patients suggesting IGF1R, alone or together with SphK1, acts as a positive prognostic indicator (Table 3, *p* = 0.018, Fig. 2a and *p* = 0.028,; Fig. 2b, respectively). These highly significant effects on OS were not seen for DFS (data not shown). No significant relationship between SphK1 expression alone and OS was identified (Table 3). In contrast, no significant improvement in OS was detected in patients with high p-IGF1R protein expression alone or combined with SphK1 in our patient analysis (Table 3, *p* = 0.303, Fig. 2c and *p* = 0.118, Fig. 2d, respectively). However, pIGF1R and SphK1 high co-expression did lead to lower *p*-value compared to either protein in isolation (Table 3). After stratification for ER status we found, in line with previous studies, an improved OS in ER-positive breast cancer patients who express high IGF1R protein in isolation (Table 3, *p* = 0.048, Fig. 3a) or combined with SphK1 (Table 3, *p* = 0.051, Fig. 3b), compared to ER-negative breast cancers (Table 3, *p* = 0.445, Fig. 3c and *p* = 0.582, Fig. 3d, respectively). This effect was further supported by a strong positive correlation between IGF1R and SphK1 in ER-positive (*p* = 0.001), but not ER-negative (*p* = 0.936) breast cancer patients (Table 4). In support of the literature reporting that pIGF1R can be detected in the nucleus of the cell, bind DNA and act as a transcription factor, we also found that some patient samples showed positive nuclear IHC staining for pIGF1R (Additional file 2) [34].

**Table 3:** Summary of Kaplan-Meier Analyses

Prognostic Marker	Log-rank p-value	Total number	Events
Non-Stratified			
IGF1R	0.018*	233	19
SphK1	0.427	233	19
IGF1R/SphK1	0.028*	232	19
pIGF1R	0.303	232	19
pIGF1R/SphK1	0.118	232	19
ER-positive			
IGF1R	0.048*	165	11
SphK1	0.250	165	11
IGF1R/SphK1	0.051	165	11
ER-negative			
IGF1R	0.445	68	8
SphK1	0.675	68	8
IGF1R/SphK1	0.582	68	8
Anti-Endocrine Therapy (AET)			
AET-positive vs. AET-negative	0.006*	190	17
AET-positive			
IGF1R	0.028*	190	17
SphK1	0.101	190	17
IGF1R/SphK1	0.034*	190	17
HER2-negative			
IGF1R	0.074	143	4
SphK1	0.052	143	4
pIGF1R	0.273	142	4
HER2-positive			
IGF1R	0.195	86	14
SphK1	0.062	86	14
pIGF1R	0.858	86	14
PR-negative			
IGF1R	0.290	81	10
SphK1	0.296	81	10
pIGF1R	0.410	81	10
PR-positive			
IGF1R	0.101	147	8
SphK1	0.293	147	8
pIGF1R	0.588	146	8

High vs. low prognostic marker expression *p-value significant; ≤0.05

**Table 4:** Spearman Correlation Analysis (*n* = 236)

Variables	Spearman Coefficient	*p*-value
IGF1R		
p-IGF1R	0.076	0.245
SphK1	0.139	0.033*
IGF1R (ER + ve)		
p-IGF1R	0.001	0.993
SphK1	0.261	0.001*
IGF1R (ER-ve)		
cp-IGF1R	0.126	0.295
SphK1	0.01	0.936
P-IGF1R		
SphK1	0.054	0.412
Age ≥ 51y		
p-IGF1R	0.036	0.580
IGF1R	−0.147	0.025*
SphK1	0.103	0.115
Grade		
p-IGF1R	−0.189	0.004*
IGF1R	−0.35	0.591
SphK1	0.083	0.202
ER Status		
p-IGF1R	0.177	0.007*
IGF1R	0.240	0.000*
SphK1	−0.227	0.000*
PR Status		
p-IGF1R	0.117	0.078
IGF1R	0.269	0.000*
SphK1	−0.192	0.003*
HER2 Status		
p-IGF1R	−0.206	0.002*
IGF1R	0.046	0.487
SphK1	−0.110	0.095

*p-value significant; ≤0.05

### Association between IGF1R and SphK1 expression, and ER, PR, HER2, tumor grade, and age

After stratification of tumors as either HER2 +/− or PR +/−, there was no significant prognostic effect of IGF1R (Additional file 3A-D) or p-IGF1R (Additional file 4A-D) in isolation, or combined with SphK1 expression (data not shown), in our patient cohort. Similarly, no significance was observed for high vs. low SphK1 protein expression alone following stratification for HER2 or PR (Table 3). However, as shown in Table 4, a significant association was observed between both p-IGF1R and IGF1R expression and ER-positivity (*p* = 0.007 and *p* < 0.001, respectively), and between IGF1R expression and PR-positivity (p < 0.001). Furthermore, p-IGF1R expression was negatively correlated to HER2-positive expression (Table 4, *p* = 0.002), yet this was not evident for IGF1R expression. Moreover, SphK1 high expression was inversely associated with both ER-positivity (*p* < 0.001) and PR-positivity (*p* = 0.003) in breast tumors (Table 4) which supports literature findings [35]. We were not able to identify any association between IGF1R or SphK1 and tumor grade; however, p-IGF1R was inversely correlated to tumor grade in our analysis (Table 4, *p* = 0.004). Lastly, we identified an inverse correlation of IGF1R expression to following stratification for age (i.e. ≥51y; average age of menopause) in our analysis (Table 4, *p* = 0.025).

To determine the combined effect of the IHC measurements and hormone receptor status on patient survival we undertook multivariate Cox regression (proportional hazards) analysis, using the forward stepwise (likelihood ratio) method. Analyzing the influence of IGF1R, p-IGF1R and SphK1 alone on overall patient survival, only IGF1R was significant (Table 5; *p* = 0.008). None of these variables was significant for DFS (Table 5). When ER and PR status were added to the model, IGF1R remained the only significant variable (Table 5: *p* = 0.010 for OS, not significant for DFS). However, adding ER, PR and HER status to the model, HER2 became the dominant variable when OS was the survival endpoint (Table 5; p < 0.001), with IGF1R expression approaching significance (Table 5; *p* = 0.053). For DFS, only HER2 was significant among the six variables (Table 5; *p* = 0.007).

**Table 5:** Cox-regression Multivariate Analysis

Endpoint	Cases	Events	Variables	*p*-value
OS	225	18	IGF1R	0.010*
			pIGF1R	0.330
			SphK	0.617
			ER	0.561
			PR	0.167
DFS	219	18	IGF1R	0.880
			pIGF1R	0.622
			SphK	0.305
			ER	0.470
			PR	0.128
OS	221	17	IGF1R	0.053(*)
			pIGF1R	0.544
			SphK	0.607
			ER	0.608
			PR	0.143
			HER2	0.000*
DFS	215	17	IGF1R	0.794
			pIGF1R	0.732
			SphK	0.429
			ER	0.866
			PR	0.254
			HER2	0.007*

Cases available in analysis

Method = Forward Stepwise (Likelihood Ratio)

### IGF1R and SphK1 co-expression is associated with improved disease outcome in anti-endocrine therapy treated breast cancer patients

**Fig. 4:**
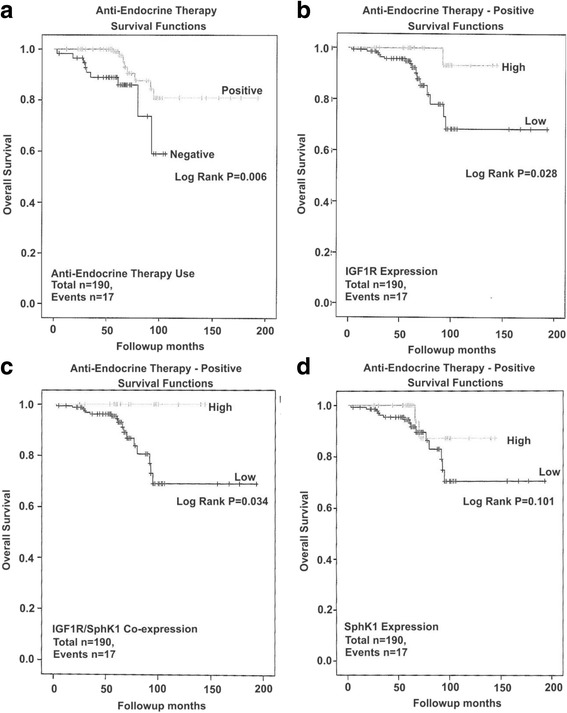
Survival outcomes in relation to IGF1R and/or SphK1 protein expression in hormone therapy treated breast cancer patients. Kaplan-Meier analysis was performed to measure the overall survival (OS) for (**a**). hormone therapy (anti-endocrine therapy) treatment (non-stratified) and further stratified for high vs. low (**b**). IGF1R (**c**). IGF1R and SphK1 co-expression and (**d**). SphK1 expression as described under Methods. Statistical significance was accepted as a log-rank *p*-value <0.05

Given that we identified a relationship between high IGF1R protein expression alone and combined with high SphK1 expression, and overall patient survival, that was most evident for ER-positive breast cancers, we undertook further analysis to determine whether a similar relationship existed when samples were stratified for anti-endocrine therapy. KM analysis identified that anti-endocrine therapy is associated with an improved OS (Table 3, *p* = 0.006, Fig. 4a) in the unstratified cohort. Examining only samples from anti-endocrine therapy treated women, high IGF1R expression (Table 3, *p* = 0.028, Fig. 4b), and high IGF1R/SphK1 co-expression (Table 3, *p* = 0.034, Fig. 4c) were both prognostic for improved OS, whereas high SphK1 expression was not (Table 3, *p* = 0.101, Fig. 4d). This supports our previous findings that high IGF1R/SphK1 co-expression is associated with an improved disease outcome specifically in ER-positive breast cancer patients in relation to anti-endocrine therapy.

### Co-targeting IGF1R and SphK1 acts synergistically on breast cancer cell viability and colony formation

**Fig. 5:**
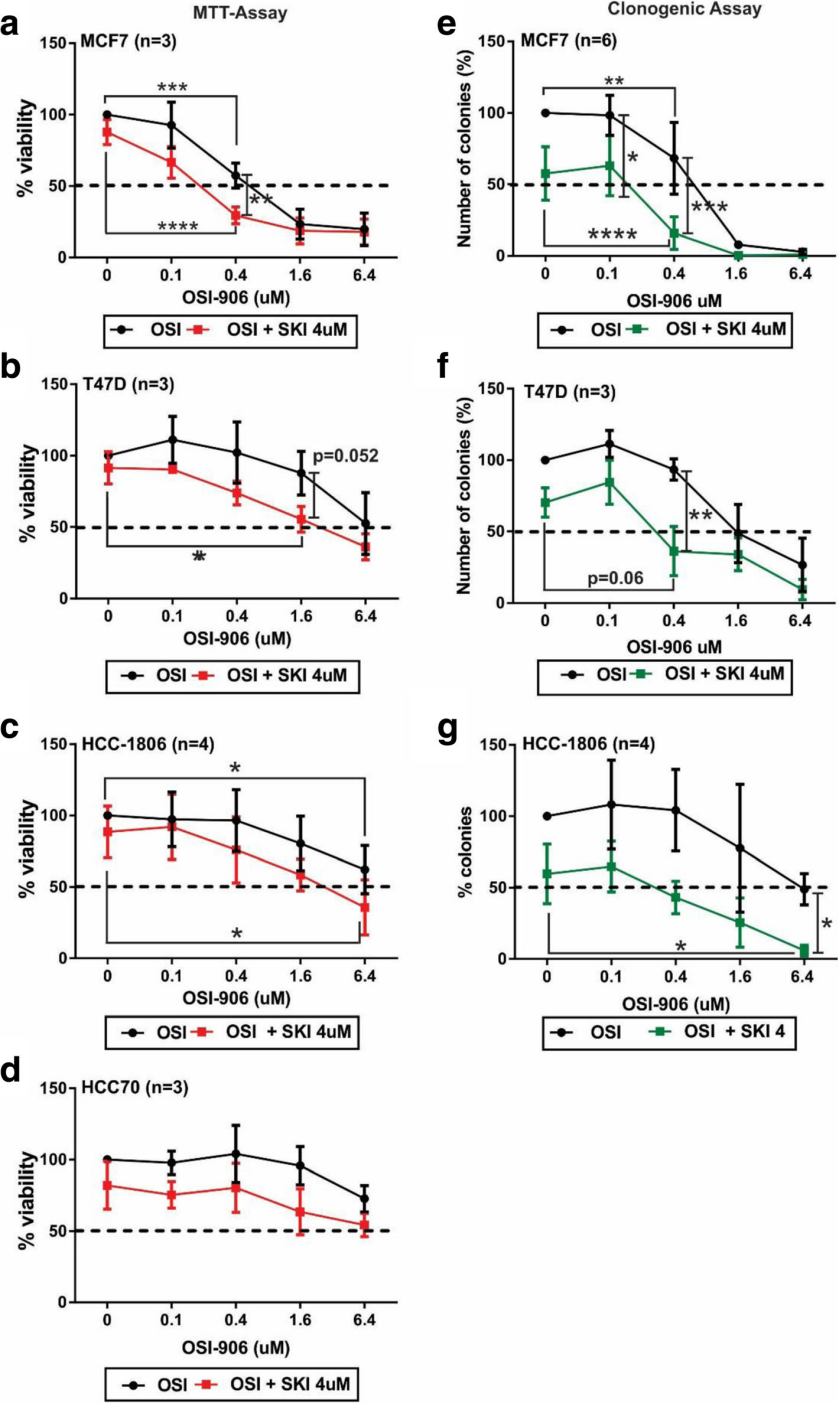
Co-targeting IGF1R and SphK1 effects on cell viability and colony formation in breast cancer cells. **a**-**d**. MTT-assay and (**e**-**g**). colony formation experiments were performed using the ER-positive; MCF7 and T47D and ER-negative; HCC1806 and HCC70 breast cancer cell-lines. 2 × 10^3^ cells were plated in either 96-well or 6-well plates, cultured for 24 h and subsequently treated with the dual IGF1R/InsR tyrosine kinase inhibitor (OSI-906; 0.1-6.4 μM) and/or SphK1inhibitor (SKI-II; 4 μM) for 96 h (MTT-assay) and 10-14 d (clonogenic assay). Repeated measures ANOVA was performed to determine the effect of SKI-II addition to OSI-906 does-response curves. Graphs depict experimental data normalized to zero treatment vehicle control and 1-way ANOVA followed by Tukey’s test was performed to determine significance between treatment groups and significance accepted *p*-values *p < 0.05, **p < 0.01, ***p < 0.001 and ****p < 0.0001. All experiments were performed in triplicate for MTT-assay and duplicate for clonogenic assay. Note: The SKI 4 μM plus OSI 6.4 μM treatment was only performed in duplicate for the HCC1806 clonogenic assay experiments

The observation that high IGF1R and SphK1 expression are prognostic for improved overall survival is paradoxical, given that the literature suggests IGF1R and SphK1 are oncogenic mediators in breast cancer. Since the positive prognostic effect was only significant for ER-positive cancers, we undertook in vitro studies to compare whether therapeutically co-targeting IGF1R and SphK1 using the dual IGF1R/InR inhibitor OSI-906 and the SphK inhibitor SKI-II might be a viable clinical approach for ER-negative vs. ER-positive cancers. To determine optimal dose ranges to identify a combination effect of the two drugs, initial MTT-assay experiments were performed on each drug alone, i.e. OSI-906 at 0.1, 0.4, 1.6 and 6.4 μM (Fig. 5) and SKI-II at 0.16, 0.8, 4, 10 and 20 μM (Additional file 5), as well as all combinations of these doses (data not shown). Using immunoblot analysis we confirmed inhibition of p-IGF1R phosphorylation and downstream IGF1R signaling (i.e. p-AKT and 4E-BP1 hyper-phosphorylation) following treatment with OSI-906 and SphK1 steady-state protein levels by SphK1 in the MCF7 ER-positive and HCC1806 ER-negative cell-line to demonstrate effective drug-target inhibition by the therapies (Additional file 6). Based on the initial MTT-experiments we identified that a fixed concentration of SKI-II at 4 μM in combination with OSI-906 in the range of 0.1-6.4 μM was the most effective in reducing cell viability (Fig. 5).

OSI-906 showed dose-dependent inhibition of the ER-positive MCF7 breast cancer cell line by both cell-viability and colony-formation assays, with a near-maximal effect seen at 1.6 μM (Fig. 5a and e). The addition of 4 μM SKI-II significantly increased sensitivity to OSI-906 by 2- to 4-fold in both assays (*p* < 0.001 by repeated measures ANOVA for both assays), with the greatest effect of the drug combination, compared to individual treatments, seen at 4 μM SKI-II and 0.4 μM OSI-906 (Fig. 5a; *p* < 0.01 and Fig. 5e; p < 0.001, and Additional file 7A). The ER-positive T47D cell-line was somewhat less sensitive to OSI-906, but a similar sensitising effect of 4 μM SKI-II was seen (Fig. 5b and f; p < 0.001 by repeated measures ANOVA for both assays). Greatest combination effects, compared to individual treatments, were seen at 4 μM SKI-II and 1.6 μM OSI-906 co-treatment in the cell-viability assay (Fig. 5f; *p* = 0.052), and at 4 μM SKI-II and 0.4 μM OSI-906 co-treatment in the colony-formation assay (Fig. 5f; p < 0.01 and Additional file 7B).

The ER-negative HCC1806 cell line showed similar OSI-responsiveness to T47D, and similarly, the addition of 4 μM SKI-II significantly increased responsiveness (Fig. 5c and g; *P* < 0.001 by repeated measures ANOVA for both assays). The greatest combination effect of SKI-II and OSI-906 co-treatment, compared to individual treatments, was observed on colony formation at the highest dose of 6.4 μM OSI-906 (Fig. 5g; *p* < 0.05 and Additional file 7C). ER-negative HCC70 cells were highly resistant to inhibition by OSI-906 (Fig. 5d), and even in the presence of 4 μM SKI-II failed to achieve 50% inhibition of cell viability at the highest tested OSI-906 dose of 6.4 μM. HCC70 cells expressed low levels of p-IGF1R and SphK1 protein (data not shown) which is likely a contributing factor to the lack of co-treatment effectiveness.

Calculation of the Chou-Talalay combination index confirmed our findings that co-treatment with OSI-906 and SKI-II led to synergistic effects using OSI-906 at 0.4 μM combined with SKI-II at 4 μM on cell viability and/or colony formation in the MCF7 and T47D ER-positive cell-lines, and at the higher dose of OSI-906 at 6.4 μM in the HCC1806 ER-negative cell-line (i.e. CI < 1; synergism; Table 6). Drug synergy could not be calculated for HCC70 cells owing to their strong resistance to OSI-906.

**Table 6:** Chou Talalay Drug Synergism (Combination Index)

MCF7 (ER+)	CI (mean ± SD)
4 μM SKI-II + 0.4 μM OSI-906 – MTT Assay	0.49 ± 0.40 (*n* = 3)
4 μM SKI-II + 0.4 μM OSI-906 – Clonogenic Assay	0.38 ± 0.28 (*n* = 6)
T47D (ER+)	CI (mean ± SD)
4 μM SKI-II + 0.4 μM OSI-906 – Clonogenic Assay	0.21 ± 0.05 (*n* = 3)
HCC1806 (ER-)	CI (mean ± SD)
4 μM SKI-II + 6.4 μM OSI-906 – Clonogenic Assay	0.01 ± 0.01 (*n* = 2)

*Abbreviation*: *CI* Combination Index

## Discussion

Given that IGF1R is a well-documented oncogenic factor in many different types of cancers, and therapeutically targeting its activity in isolation has proven to be unsuccessful, a better understanding of its signaling activity is certainly required [6]. Clinically ineffective IGF1R-directed therapies may be associated with the ability of the cancer cell to re-activate the signaling pathway in addition to alternative activation of downstream IGF1R signaling pathways by other growth-promoting receptors in the cell such as HER2/3, EGFR and InsR [12, 36]. This study has provided novel findings demonstrating that the high co-expression of IGF1R and SphK1 may have positive prognostic significance for overall (but not disease free) survival in ER-positive breast cancer, but in line with pre-clinical findings demonstrating IGF1R and SphK1 can promote oncogenic effects in cancer, paradoxically may still be considered for therapeutic targeting [6].

We have recently reported that high IGF1R and SphK1 gene co-expression, based on data derived from public databases, is associated with a worse survival outcome in ER-positive, but not ER-negative breast cancer [6]. However, our immunohistochemistry findings in this study report the opposite effect for protein expression (i.e. improved OS in ER-positive disease), further highlighting the ongoing discrepancy in findings relating to the relationship between high IGF1R expression and ER-positive breast cancer potentially based on different prognostic parameters (i.e. protein vs gene, patient tumor cohorts, antibodies, cut-off and pIGF1R vs. IGF1R measurements i.e. signalling vs. expression) [12]. In light of the differences between protein versus gene expression data, consideration of whether measurement of gene expression is an accurate indicator of clinical outcomes in women with breast cancer requires further investigation. Importantly, mRNA expression levels have been shown to be a poor predictor of protein levels and in fact translational control is a more accurate predictor of protein expression [7, 37], highlighting that protein abundance rather than mRNA expression is likely to be a more accurate predictor of patient outcomes. Furthermore, expression of IGF1R vs. activity of the signaling pathway (i.e. pIGF1R and activation of IGF1R signaling) play an integral oncogenic role in cancer and likely add to the discrepancy between studies and analysis (i.e. gene vs protein) reporting the prognostic and therapeutic significance of whether blocking IGF1R activity and/or expression is more beneficial in cancer [12].

In this study p-IGF1R was not a prognostic factor yet Kaplan-Meier analyses revealed that p-IGF1R and SphK1 high co-expression did improve the log-rank *p*-value compared to either protein in isolation, suggesting that the activity of p-IGF1R signaling may be important and supporting the literature that active IGF1R signaling leads to upregulation of SphK1 expression [38, 39]. Furthermore, we did detect a significant positive correlation of both p-IGF1R and IGF1R to both ER expression and IGF1R to PR expression [40], and p-IGF1R was additionally negatively correlated to HER2 expression. Nevertheless, high p-IGF1R expression has been reported to be associated with a worse survival outcome in breast cancer [10] and based on the literature and this study, there are clearly some conflicting data in relation to whether IGF1R and/or p-IGF1R is the more useful breast cancer prognostic marker. Since pIGF1R can be detected also in the nucleus of the cell and act as a transcription factor, the prognostic significance of membranous/cytoplasmic vs. nuclear staining may be of clinical significance and needs to be further investigated [27] Quantitation of p-IGF1R is potentially confounded by pre-analytical factors such as tissue storage and processing times, since active phosphatases have the ability to cause uncontrolled dephosphorylation. This issue is avoided by the measurement of total IGF1R.

In line with improved therapeutic outcomes associated with ER-directed cancer therapies in ER-positive versus ER-negative breast cancers, our study supports the notion that individuals who receive anti-endocrine targeted-therapies (i.e. diagnosed as ER-positive) have a much better outcome than non-anti-endocrine targeted-therapies [41]. Moreover, a better prognosis has been shown to be associated with non-triple-negative versus triple-negative breast cancer [42], as expected since ER-positive disease at present has better clinical management. IGF1R mRNA has been reported as a good prognostic factor specifically in luminal breast cancer subtypes which was also correlated to IGF1R protein expression [9, 40, 43]. However, within the luminal A subtype, high IGF1R mRNA expression is associated with a worse patient outcome than low expression and has been suggested to be a contributing factor to anti-estrogen therapy resistance [41].

Based on the literature linking both IGF1R and SphK1 to ER-positive breast cancer, we also demonstrated that IGF1R and SphK1 are positively correlated in ER-positive but not ER-negative disease. Our findings support the literature reporting that higher SphK1 expression is evident in ER-negative breast cancer (i.e. inverse correlation of SphK1 to ER status), compared to ER-positive disease [35]. This finding was also extended to PR status, which supports the biology of PR as an ER-regulated gene [44]. Interestingly we further detected an inverse correlation of IGF1R to age (i.e. average age of menopausal status) and p-IGF1R to tumor grade and HER2 status. Moreover, IGF1R expression is reported to be significantly associated to HER2-positivity and poorer disease-free survival in premenopausal women, suggesting a link between IGF1R and HER2 specifically in pre-menopausal breast cancer [45]. In this context it is notable that multivariate survival analysis on our predominantly post-menopausal cohort found that high IGF1R expression was the only significant variable associated with overall survival if HER2 status was excluded from the model, but that HER2 status assumed the dominant effect when added to the analysis, with high IGF1R expression as the only other measured factor approaching significance. This may indicate that the variable findings in the literature in regard to the prognostic value of IGF1R measurement could be influenced by concomitant HER2 status, and suggests that further examination of the effects of IGF1R and HER2 co-expression is warranted. Serum IGF-1 has also been reported to be associated with mammographic density in pre-menopausal women and this may be linked to IGF1R activity [46].

It is well accepted that IGF1R can regulate ER, and SphK1 is regulated by estrogen, in breast cancer [13, 47, 48], hence the therapeutic implication that co-targeting them may be clinically effective in ER-positive breast cancer. Despite the lack of patient survival significance in relation to high IGF1R and Sphk1 co-expression in our ER-negative patient cohort, IGF1R and SphK1 are expressed in ER-negative cells and may have therapeutic significance [35, 49]. Our findings show that, despite high IGF1R and/or SphK1 being associated with a positive clinical outcome in ER-positive breast cancer while having no effect in ER-negative breast cancer, IGF1R and SphK1 co-targeting is worth investigating for clinical benefit in these women. The molecular basis to this finding may be associated with the improvement in anti-endocrine therapy in women who express IGF1R and SphK1. Further prognostic studies using a recurrent disease population vs. non-recurrent ER-positive breast cancer population would be helpful since both IGF1R and SphK1 are reported to contribute to tamoxifen resistance [16, 25, 26]. Moreover, since both IGF1R and SphK1 are reported to be associated with anti-estrogen therapy resistance [16, 25, 26], it is important that future studies using IGF1R-targeted therapies which have not been successful as monotherapies also investigate the benefit of including other IGF1R co-related factors such as SphK1 as targets.

We conducted in vitro studies in ER-positive and -negative cell lines in an attempt to dissect the different prognostic potential of IGF1R expression in ER-positive and –negative cancers. The two ER-negative cell lines tested were both more resistant to OSI-906 than the ER-positive lines, but the synergistic co-inhibition of cell viability by IGF1R and SphK1 inhibition was at least as effective in the ER-negative line HCC1806 as in the two ER-positive lines, providing a possible rationale for evaluating a combination of IGF1R and SphK1 inhibitors in vivo, particularly in ER-negative tumors. IGF1R signaling can occur in response to the formation of either a homodimer or a hybrid receptor (i.e. heterodimer) with the InsR. Since OSI-906 is a dual IGF1R/InsR inhibitor and both receptors are potential therapeutic targets in breast cancer [36] it is important to consider the inhibitory contribution of both receptors to OSI-906 effects. However, since we did not use a totally selective InsR inhibitor or measure changes to p-InsR it is difficult to ascertain the contribution of OSI-906 mediated inhibition of InsR vs IGF1R. Nevertheless, we have shown that OSI-906 reduces both p-IGF1R steady-state protein and IGF1R signaling (i.e. p-AKT and 4E-BP-1 hyper-phosphorylation) in the MCF7 ER-positive and HCC1806 ER-negative breast cancer cell-lines, consistent with the assumption that the therapeutic inhibition of OSI-906 is mediated at least in part via IGF1R.

In this study we have provided new evidence demonstrating a prognostic relationship between IGF1R and the co-related SphK1 signaling pathway in relation to: 1) ER, PR and HER2 status and 2) anti-endocrine targeted therapies. Moreover, our preclinical findings suggest that co-targeting IGF1R and SphK1 may have benefit in some women with breast cancer and requires further investigation.

## Conclusion

In conclusion the findings in this study support the proposal that high tissue IGF1R levels, alone and in combination with high SphK1 levels, are prognostic indicators in ER-positive breast cancer and in response to anti-endocrine therapy. Furthermore, since IGF1R is an established oncogenic factor in breast cancer and has been the focus of numerous clinical trials, it is potentially of therapeutic importance that we have shown that co-targeting both IGF1R and SphK1 in vitro has a synergistic benefit. There is an ongoing need to understand how to effectively target IGF1R in breast cancer and to identify new IGF1R-related co-factors for clinical intervention. This study may provide a better understanding of IGF1R-signaling and help to explain the lack of clinical outcome benefit of IGF1R-directed monotherapies in breast cancer.

## Additional files

Additional file 1: Table S1. Full dataset used in survival analyses. (PDF 86 kb)

Additional file 2: Immunohistochemistry and manual scoring analysis of Australian Breast Cancer Tissue Bank patient samples. Refer to Fig. 1 for experimental details. (DOCX 332 kb)

Additional file 3: Prognostic survival outcomes in relation to IGF1R protein expression in HER2 and PR-positive and negative breast cancer tissues. Kaplan-Meier analysis was performed to measure the overall survival (OS) following stratification for high vs. low IGF1R protein expression, stratified for HER2 and PR expression: **A.** IGF1R (HER2-negative); **B.** IGF1R (HER2-positive); **C.** IGF1R (PR-negative) and **D.** IGF1R (PR-negative) as described under Methods. (DOCX 264 kb)

Additional file 4: Survival outcomes in relation to p-IGF1R protein expression in HER2 and PR-positive and negative breast cancer tissues. Kaplan-Meier analysis was performed to measure the overall survival (OS) following stratification for high vs. low p-IGF1R protein expression, stratified for HER2 and PR expression: **A.** p-IGF1R (HER2-negative); **B.** p-IGF1R (HER2-positive); **C.** p-IGF1R (PR-negative) and **D.** p-IGF1R (PR-negative) as described under Methods. (DOCX 279 kb)

Additional file 5: Breast cancer cell viability dose curves in response to SKI-II. Refer to Fig. 5 for experimental details. **A.** MCF7, **B.** T47D, **C.** HCC-1806 and **D.** HCC70 were treated with single agents of the SphK1 inhibitor (SKI-II; 0.16, 0.8, 4, 10 and 20 μM) for 96 h. Graphs depict experimental data normalized to zero treatment vehicle control and 1-way ANOVA followed by Tukey’s test was performed to determine significance between treatment groups and significance accepted *p*-values *p < 0.05, **p < 0.01, ***p < 0.001 and ****p < 0.0001. (DOCX 186 kb)

Additional file 6: OSI-906 inhibits p-IGF1R and IGF1R signaling factors in breast cancer cell-lines. 3 × 10^5^ ER-positive **A.** MCF7 and ER-negative **B.** HCC-1806 breast cancer cells were plated per well of a 6-well plate and cultured for 24 h and subsequently treated with the dual IGF1R/InsR dual tyrosine kinase inhibitor (OSI-906; 0.1, 0.4 and 1.6 μM) and/or SphK1 inhibitor (SKI-II; 4 μM) for 24 h. Protein lysates were collected and 20 μg of protein was used for immunoblot analysis to measure changes to IGF1R signaling (i.e. AKT and eIF4E-BP1 (protein translation)) and SphK1 steady-state protein expression levels. B-actin was used as a loading control. Abbreviations: Hyper-P = hyper-phosphorylation and Hypo-P = hypophosphorylated. (DOCX 255 kb)

Additional file 7: Effects of co-targeting IGF1R and SphK1 on colony formation in breast cancer cell-lines. Refer to Fig. 5 for experimental details. Images represent individual wells of colonies following drug treatments for 10-14 d of ER-positive **A.** MCF7 and **B.** T47D and ER-negative **C.** HCC1806 breast cancer cell-lines. Images were captured using FujiFilm Luminescent Image Analyzer LAS-300 (Stamford, CT) and single colonies were counted using open colony forming unit (CFU) software (http://opencfu.sourceforge.net/). (DOCX 545 kb)
