# Supplementary material for: Insulin-like growth factor receptor and sphingosine kinase are prognostic and therapeutic targets in breast cancer
Source: BMC Cancer. 2017 Dec 5;17:820. doi: 10.1186/s12885-017-3809-0 (PMC5718000; doi:10.1186/s12885-017-3809-0)
Supplement: Supplementary file 1 — Full dataset used in survival analyses. (PDF 86 kb) [file 12885_2017_3809_MOESM1_ESM.pdf]

Supplementary Table 1: Full dataset used in survival analyses (page 1)

Patient ID	Followup (months)	OS	BrCa Events	IGF1R Score	IGF1R bin ‡	pIGF1R Score	pIGF1R bin ‡	SphK Score	SphK bin ‡	IGF1R-SphK Comb ❖	pIGF1R-SphK Comb ❖	ER status	PR status	HER2 status	Anti-estrogen therapy (AET)	AET-IGF1R	AET-SphK	AET-IGF1R-SphK
01-06-010	106	0	0	0	0	1	0	2	1	0	0	Positive	Negative	Positive	0	0	0	0
01-06-028	80	1	1	1	0	0	0	2	1	0	0	Negative	Negative	Positive	0	0	0	0
01-06-033	96	0	0	0	0	0	0	0	0	0	0	Positive	Positive	Negative	1	0	0	0
01-06-036	100	0	0	0	0	0	0	1	0	0	0	Positive	Positive	Positive	1	0	0	0
01-06-048	104	0	0	1	0	0	0	2	1	0	0	Negative	Negative	Positive	0	0	0	0
01-06-056	103	0	0	0	0	0	0	1	0	0	0	Positive	Positive	Positive	1	0	0	0
01-06-058	74	0	0	0	0	2	1	2	1	0	1	Negative	Negative	Negative	0	0	0	0
01-06-061	61	1	0	0	0	0	0	0	0	0	0	Negative	Negative	Positive	0	0	0	0
01-06-063	101	0	0	2	1	0	0	0	0	0	0	Positive	Positive	Negative	1	1	0	0
01-06-066	95	0	0	0	0	2	1	0	0	0	0	Negative	Positive	Negative	0	0	0	0
01-06-074	95	1	0	1	0	0	0	0	0	0	0	Positive	Positive	Negative	1	0	0	0
01-06-086	97	0	0	1	0	1	0	1	0	0	0	Positive	Positive	Negative	0	0	0	0
01-06-087	101	0	0	1	0	0	0	2	1	0	0	Positive	Positive	Negative	1	0	1	0
01-06-093	96	0	0	2	1	0	0	0	0	0	0	Positive	Positive	Negative	✖	✖	✖	✖
01-06-099	88	0	0	1	0	0	0	0	0	0	0	Positive	Positive	Negative	1	0	0	0
01-06-100	56	1	1	0	0	0	0	0	0	0	0	Positive	Positive	Positive	1	0	0	0
01-06-105	28	1	1	0	0	0	0	1	0	0	0	Negative	Negative	Positive	✖	✖	✖	✖
01-06-106	18	1	1	1	0	0	0	2	1	0	0	Negative	Positive	Positive	0	0	0	0
01-06-110	95	0	0	1	0	1	0	3	1	0	0	Negative	Negative	Negative	✖	✖	✖	✖
01-06-111	97	0	0	2	1	0	0	2	1	1	0	Positive	Positive	Negative	1	1	1	1
01-07-003	73	0	0	1	0	0	0	1	0	0	0	Negative	Positive	Positive	0	0	0	0
01-07-019	61	0	0	0	0	0	0	3	1	0	0	Negative	Negative	Negative	0	0	0	0
01-07-025	55	0	0	1	0	1	0	2	1	0	0	Negative	Negative	Positive	0	0	0	0
01-07-028	62	0	0	1	0	3	1	2	1	0	1	Positive	Positive	Positive	1	0	1	0
01-07-029	65	0	0	1	0	0	0	1	0	0	0	Negative	Negative	Positive	✖	✖	✖	✖
01-07-036	60	0	0	1	0	0	0	2	1	0	0	Negative	Negative	Positive	✖	✖	✖	✖
01-07-038	60	0	0	1	0	0	0	0	0	0	0	Positive	Positive	Negative	1	0	0	0
01-07-040	54	0	0	2	1	1	0	3	1	1	0	Positive	Positive	Positive	1	1	1	1
01-07-042	61	0	0	0	0	0	0	1	0	0	0	Positive	Positive	Negative	✖	✖	✖	✖
01-07-043	30	1	0	0	0	0	0	2	1	0	0	Positive	Positive	Positive	0	0	0	0
01-07-045	23	0	0	0	0	2	1	1	0	0	0	Positive	Positive	Negative	1	0	0	0
01-07-052	62	0	0	0	0	0	0	1	0	0	0	Negative	Negative	Negative	✖	✖	✖	✖
01-07-053	60	0	0	2	1	0	0	2	1	1	0	Positive	Positive	Negative	1	1	1	1
01-07-061	68	0	0	1	0	2	1	3	1	0	1	Positive	Positive	Negative	✖	✖	✖	✖
01-07-064	72	0	1	2	1	2	1	3	1	1	1	Positive	Positive	Negative	✖	✖	✖	✖
01-07-073	68	0	0	2	1	0	0	2	1	1	0	Negative	Negative	Negative	0	0	0	0
01-07-080	62	0	0	1	0	0	0	2	1	0	0	Negative	Negative	Negative	✖	✖	✖	✖
01-07-086	63	0	0	0	0	0	0	1	0	0	0	Positive	Positive	Positive	✖	✖	✖	✖
01-07-087	70	1	1	0	0	0	0	2	1	0	0	Positive	Negative	Negative	1	0	1	0
01-07-088	90	0	0	0	0	2	1	1	0	0	0	Positive	Positive	✖	1	0	0	0
01-07-091	54	0	1	1	0	0	0	1	0	0	0	Positive	Positive	Negative	✖	✖	✖	✖
01-07-092	58	0	0	0	0	0	0	1	0	0	0	Positive	Positive	Positive	✖	✖	✖	✖
01-07-095	60	0	0	1	0	0	0	2	1	0	0	Positive	Negative	Positive	✖	✖	✖	✖
01-07-099	56	0	0	1	0	1	0	2	1	0	0	Positive	Positive	Negative	✖	✖	✖	✖
01-07-106	60	0	0	0	0	0	0	0	0	0	0	Positive	Positive	Negative	✖	✖	✖	✖
01-07-111	52	0	0	1	0	0	0	0	0	0	0	Positive	Positive	Negative	1	0	0	0
01-07-112	55	0	0	0	0	0	0	1	0	0	0	Positive	Negative	Positive	1	0	0	0
01-07-117	26	0	0	1	0	1	0	0	0	0	0	Positive	Positive	Positive	1	0	0	0

Supplementary Table 1: Full dataset used in survival analyses (page 2)

Patient ID	Followup (months)	OS	BrCa Events	IGF1R Score	IGF1R bin ‡	pIGF1R Score	pIGF1R bin ‡	SphK Score	SphK bin ‡	IGF1R-SphK Comb ❖	pIGF1R-SphK Comb ❖	ER status	PR status	HER2 status	Anti-estrogen therapy (AET)	AET-IGF1R	AET-SphK	AET-IGF1R-SphK
01-07-120	53	0	0	2	1	2	1	1	0	0	0	Positive	Positive	Negative	1	1	0	0
01-07-123	56	0	0	1	0	0	0	1	0	0	0	Positive	Positive	Positive	✖	✖	✖	✖
01-08-002	64	0	0	3	1	0	0	3	1	1	0	Positive	Positive	Negative	1	1	1	1
01-08-009	28	1	0	0	0	0	0	0	0	0	0	Positive	Positive	Positive	0	0	0	0
01-08-016	63	0	0	0	0	0	0	2	1	0	0	Negative	Negative	Negative	0	0	0	0
01-08-018	60	0	0	0	0	0	0	0	0	0	0	Negative	Equivoca	Positive	0	0	0	0
01-08-022	72	0	0	3	1	0	0	2	1	1	0	Positive	Positive	Positive	1	1	1	1
01-08-023	64	0	0	1	0	0	0	3	1	0	0	Positive	Positive	Negative	1	0	1	0
01-08-024	59	0	0	1	0	0	0	2	1	0	0	Positive	Positive	Positive	1	0	1	0
01-08-026	60	0	0	0	0	1	0	2	1	0	0	Negative	Negative	Negative	0	0	0	0
01-08-033	61	0	0	2	1	0	0	1	0	0	0	Negative	Negative	Positive	0	0	0	0
01-08-039	72	0	0	1	0	0	0	2	1	0	0	Negative	Positive	Positive	0	0	0	0
01-08-055	61	0	0	0	0	0	0	2	1	0	0	Negative	Positive	Negative	✖	✖	✖	✖
01-08-057	73	0	0	0	0	2	1	2	1	0	1	Positive	Positive	Positive	0	0	0	0
01-08-059	63	0	0	0	0	2	1	3	1	0	1	Negative	Negative	Negative	0	0	0	0
01-08-060	61	0	0	0	0	0	0	2	1	0	0	Negative	Negative	Negative	✖	✖	✖	✖
01-08-084	44	0	0	1	0	0	0	2	1	0	0	Negative	Positive	Positive	1	0	1	0
01-08-090	55	0	0	1	0	0	0	3	1	0	0	Positive	Positive	Positive	1	0	1	0
01-08-101	61	0	0	1	0	0	0	2	1	0	0	Negative	Negative	Positive	0	0	0	0
01-08-102	61	0	1	2	1	0	0	2	1	1	0	Positive	Negative	Positive	0	0	0	0
01-08-108	61	0	0	2	1	0	0	2	1	1	0	Positive	Positive	Positive	1	1	1	1
01-08-112	27	0	0	0	0	0	0	2	1	0	0	Positive	Positive	Positive	0	0	0	0
01-08-120	61	0	0	1	0	0	0	3	1	0	0	Negative	Negative	Positive	0	0	0	0
01-08-138	35	1	1	0	0	0	0	2	1	0	0	Negative	Negative	Positive	0	0	0	0
01-08-142	60	0	0	2	1	1	0	3	1	1	0	Negative	Negative	Positive	0	0	0	0
01-08-148	53	0	0	1	0	0	0	2	1	0	0	Negative	Negative	Positive	0	0	0	0
01-08-152	66	1	1	0	0	0	0	2	1	0	0	Negative	Equivoca	Positive	1	0	1	0
01-08-159	50	0	0	1	0	1	0	3	1	0	0	Negative	Negative	Positive	0	0	0	0
01-09-029	59	0	0	0	0	0	0	3	1	0	0	Negative	Negative	Negative	✖	✖	✖	✖
01-09-031	59	0	0	3	1	0	0	3	1	1	0	Positive	Positive	Negative	1	1	1	1
01-09-040	26	0	0	0	0	0	0	3	1	0	0	Positive	Positive	Negative	1	0	1	0
01-09-045	61	0	0	0	0	0	0	2	1	0	0	Negative	Negative	Negative	0	0	0	0
01-09-050	61	1	0	0	0	0	0	3	1	0	0	Negative	Negative	Negative	✖	✖	✖	✖
01-09-053	70	0	0	0	0	0	0	3	1	0	0	Negative	Negative	Negative	0	0	0	0
01-09-067	137	0	0	0	0	0	0	3	1	0	0	Negative	Negative	Negative	✖	✖	✖	✖
01-09-069	8	0	0	2	1	1	0	3	1	1	0	Positive	Positive	Negative	✖	✖	✖	✖
01-09-070	66	0	0	0	0	0	0	2	1	0	0	Negative	Negative	Negative	✖	✖	✖	✖
01-09-113	37	0	0	0	0	0	0	2	1	0	0	Negative	Negative	Negative	0	0	0	0
01-09-130	63	0	0	1	0	0	0	3	1	0	0	Positive	Positive	Negative	1	0	1	0
01-09-140	65	0	0	0	0	2	1	2	1	0	1	Negative	Negative	Negative	✖	✖	✖	✖
02-07-014	60	0	0	3	1	2	1	1	0	0	0	Positive	Positive	✖	1	1	0	0
02-07-031	325	0	0	2	1	2	1	2	1	1	1	Negative	Negative	✖	0	0	0	0
02-08-006	65	0	0	0	0	0	0	3	1	0	0	Negative	Negative	Negative	0	0	0	0
02-09-021	65	0	0	1	0	2	1	3	1	0	1	Positive	Positive	Negative	1	0	1	0
02-09-037	64	0	0	1	0	1	0	3	1	0	0	Positive	Positive	Negative	0	0	0	0
02-09-050	60	0	0	0	0	2	1	2	1	0	0	Positive	Positive	Negative	0	0	0	0
02-09-066	60	0	0	0	0	2	1	3	1	0	1	Positive	Positive	Negative	1	0	1	0
03-06-004	89	0	0	0	0	0	0	1	0	0	0	Negative	Negative	Negative	0	0	0	0

Supplementary Table 1: Full dataset used in survival analyses (page 3)

Patient ID	Followup (months)	OS	BrCa Events	IGF1R Score	IGF1R bin ‡	pIGF1R Score	pIGF1R bin ‡	SphK Score	SphK bin ‡	IGF1R-SphK Comb ❖	pIGF1R-SphK Comb ❖	ER status	PR status	HER2 status	Anti-estrogen therapy (AET)	AET-IGF1R	AET-SphK	AET-IGF1R-SphK
03-06-007	98	0	0	3	1	1	0	3	1	1	0	Positive	Positive	Negative	1	1	1	1
03-06-008	100	0	0	2	1	✖	✖	3	1	1	✖	Positive	Positive	Negative	1	1	1	1
03-06-019	51	0	0	2	1	0	0	3	1	1	0	Positive	Positive	Negative	✖	✖	✖	✖
03-06-021	97	0	0	2	1	0	0	2	1	1	0	Positive	Positive	Negative	1	1	1	1
03-06-022	95	0	0	2	1	0	0	3	1	1	0	Positive	Positive	Negative	✖	✖	✖	✖
03-06-024	58	0	1	2	1	1	0	1	0	0	0	Positive	Positive	Negative	1	1	0	0
03-06-026	92	0	0	1	0	1	0	0	0	0	0	Positive	Positive	Positive	1	0	0	0
03-06-028	88	0	0	0	0	0	0	1	0	0	0	Positive	Positive	Negative	1	0	0	0
03-06-033	96	0	0	1	0	0	0	0	0	0	0	Positive	Positive	Positive	1	0	0	0
03-06-035	96	0	0	2	1	0	0	2	1	1	0	Positive	Positive	Negative	1	1	1	1
03-06-036	97	0	0	3	1	0	0	2	1	1	0	Positive	Positive	Negative	1	1	1	1
03-06-037	93	0	0	2	1	1	0	0	0	0	0	Positive	Positive	Negative	1	1	0	0
03-06-039	92	0	0	1	0	0	0	3	1	0	0	Negative	Negative	Negative	✖	✖	✖	✖
03-06-042	158	0	0	1	0	1	0	2	1	0	1	Negative	Negative	Positive	✖	✖	✖	✖
03-06-043	96	0	0	2	1	1	0	1	0	0	0	Positive	Positive	Negative	1	1	0	0
03-06-046	96	0	0	2	1	0	0	3	1	1	0	Positive	Positive	Negative	1	1	1	1
03-06-048	54	0	0	2	1	0	0	0	0	0	0	Positive	Positive	Negative	1	1	0	0
03-06-049	5	0	0	1	0	0	0	0	0	0	0	Negative	Negative	Negative	0	0	0	0
03-06-050	95	0	0	1	0	0	0	0	0	0	0	Positive	Positive	Negative	1	0	0	0
03-06-052	92	1	1	3	1	2	1	1	0	0	0	Positive	Positive	✖	1	1	0	0
03-06-053	95	0	0	3	1	2	1	1	0	0	0	Positive	Negative	Negative	1	1	0	0
03-06-055	77	1	1	1	0	0	0	1	0	0	0	Positive	Positive	Negative	1	0	0	0
03-07-001	70	0	0	0	0	0	0	0	0	0	0	Positive	Positive	Negative	1	0	0	0
03-07-002	62	0	0	0	0	0	0	1	0	0	0	Positive	Positive	Negative	1	0	0	0
03-07-005	131	0	0	0	0	3	1	2	1	0	1	Positive	Negative	Negative	✖	✖	✖	✖
03-07-006	31	1	1	1	0	0	0	1	0	0	0	Positive	Negative	Positive	0	0	0	0
03-07-007	66	0	0	0	0	0	0	0	0	0	0	Positive	Positive	Negative	1	0	0	0
03-07-008	69	0	0	0	0	2	1	0	0	0	0	Positive	Positive	Negative	0	0	0	0
03-07-009	68	0	0	0	0	0	0	1	0	0	0	Positive	Equivoca	Negative	1	0	0	0
03-07-011	60	0	0	0	0	1	0	0	0	0	0	Positive	Positive	Negative	1	0	0	0
03-07-012	63	0	0	0	0	0	0	0	0	0	0	Positive	Negative	Negative	1	0	0	0
03-07-014	65	0	0	1	0	0	0	0	0	0	0	Positive	Negative	Positive	1	0	0	0
03-07-015	177	0	0	1	0	1	0	0	0	0	0	Positive	Positive	Negative	1	0	0	0
03-07-016	61	0	0	0	0	0	0	1	0	0	0	Positive	Positive	Negative	1	0	0	0
03-07-019	64	0	0	0	0	2	1	0	0	0	0	Positive	Negative	Negative	1	0	0	0
03-07-020	60	0	0	0	0	0	0	0	0	0	0	Positive	Positive	Negative	✖	✖	✖	✖
03-07-022	59	0	0	2	1	0	0	0	0	0	0	Positive	Positive	Negative	1	1	0	0
03-07-024	47	0	0	0	0	2	1	0	0	0	0	Positive	Positive	Negative	1	0	0	0
03-07-026	63	0	0	1	0	0	0	0	0	0	0	Positive	Positive	Positive	1	0	0	0
03-07-029	66	0	0	2	1	0	0	1	0	0	0	Negative	Negative	Negative	0	0	0	0
03-07-031	66	0	0	1	0	0	0	0	0	0	0	Positive	Positive	Negative	1	0	0	0
03-07-032	60	0	0	1	0	1	0	0	0	0	0	Positive	Positive	Positive	1	0	0	0
03-07-033	59	0	0	0	0	0	0	0	0	0	0	Positive	Positive	Negative	1	0	0	0
03-07-036	63	0	0	0	0	0	0	1	0	0	0	Positive	Negative	Negative	1	0	0	0
03-07-040	71	0	0	0	0	0	0	0	0	0	0	Positive	Positive	Positive	1	0	0	0
03-07-042	63	0	0	0	0	1	0	0	0	0	0	Positive	Positive	Negative	1	0	0	0
03-07-044	63	0	0	0	0	0	0	0	0	0	0	Positive	Negative	Negative	1	0	0	0
03-07-048	62	0	0	1	0	1	0	1	0	0	0	Positive	Positive	Positive	1	0	0	0

Supplementary Table 1: Full dataset used in survival analyses (page 4)

Patient ID	Followup (months)	OS	BrCa Events	IGF1R Score	IGF1R bin ‡	pIGF1R Score	pIGF1R bin ‡	SphK Score	SphK bin ‡	IGF1R-SphK Comb ❖	pIGF1R-SphK Comb ❖	ER status	PR status	HER2 status	Anti-estrogen therapy (AET)	AET-IGF1R	AET-SphK	AET-IGF1R-SphK
03-07-049	61	0	0	0	0	0	0	0	0	0	0	Positive	Positive	Negative	1	0	0	0
03-07-054	60	0	0	2	1	0	0	1	0	0	0	Positive	Positive	Negative	1	1	0	0
03-07-055	61	0	0	0	0	1	0	1	0	0	0	Positive	Positive	Negative	1	0	0	0
03-07-059	61	0	0	0	0	0	0	1	0	0	0	Negative	Negative	Negative	0	0	0	0
03-07-062	60	0	0	2	1	0	0	0	0	0	0	Positive	Positive	Negative	1	1	0	0
03-07-063	61	0	0	1	0	0	0	2	1	0	0	Positive	Positive	Negative	1	0	1	0
03-07-066	140	0	0	2	1	1	0	2	1	1	0	Positive	Positive	Negative	1	1	1	1
03-07-067	193	0	0	0	0	2	1	1	0	0	0	Positive	Negative	Negative	1	0	0	0
03-07-069	55	0	0	0	0	2	1	1	0	0	0	Positive	Positive	Negative	1	0	0	0
03-07-074	56	0	0	0	0	2	1	0	0	0	0	Positive	Positive	Negative	1	0	0	0
03-07-075	156	0	0	1	0	1	0	0	0	0	0	Positive	Positive	✖	1	0	0	0
03-07-077	52	0	0	2	1	1	0	2	1	1	0	Positive	Positive	Negative	0	0	0	0
03-07-078	60	0	0	1	0	1	0	2	1	0	0	Positive	Positive	Negative	1	0	1	0
03-07-079	57	0	0	0	0	2	1	2	1	0	1	Positive	Positive	Negative	1	0	1	0
03-07-081	61	0	0	1	0	2	1	2	1	0	1	Positive	Negative	Negative	1	0	1	0
03-07-083	62	1	1	1	0	2	1	0	0	0	0	Positive	Negative	Positive	1	0	0	0
03-07-089	59	0	0	1	0	2	1	2	1	0	1	Positive	Positive	Negative	1	0	1	0
03-07-093	55	0	0	1	0	0	0	2	1	0	0	Positive	Positive	Negative	1	0	1	0
03-07-095	119	0	1	2	1	0	0	2	1	1	0	Positive	Positive	Negative	1	1	1	1
03-07-101	57	0	0	1	0	0	0	1	0	0	0	Positive	Negative	Positive	✖	✖	✖	✖
03-07-102	61	0	0	3	1	0	0	2	1	1	0	Positive	Negative	Negative	✖	✖	✖	✖
03-07-105	56	0	0	1	0	1	0	2	1	0	0	Positive	✖	Negative	✖	✖	✖	✖
03-07-106	59	0	0	0	0	0	0	3	1	0	0	Positive	Positive	Negative	1	0	0	0
03-07-110	50	0	0	0	0	0	0	1	0	0	0	Positive	Positive	Negative	1	0	0	0
03-07-112	58	0	0	1	0	1	0	2	1	0	0	Positive	Negative	Positive	1	0	1	0
03-07-113	61	0	0	0	0	0	0	2	1	0	0	Positive	Positive	Negative	✖	✖	✖	✖
03-07-115	53	0	0	0	0	0	0	2	1	0	0	Negative	Negative	Negative	0	0	0	0
03-07-116	27	0	0	2	1	0	0	1	0	0	0	Positive	Positive	Positive	1	1	0	0
03-07-117	57	0	0	0	0	1	0	0	0	0	0	Negative	Negative	Positive	0	0	0	0
03-07-118	50	0	0	1	0	2	1	0	0	0	0	Positive	Positive	Negative	1	0	0	0
03-07-120	61	0	0	1	0	0	0	1	0	0	0	Positive	Positive	Positive	1	0	0	0
03-07-121	55	0	0	1	0	3	1	0	0	0	0	Positive	Positive	Negative	1	0	0	0
03-08-008	93	1	1	0	0	0	0	1	0	0	0	Negative	Negative	Positive	0	0	0	0
03-08-009	59	0	0	2	1	2	1	0	0	0	0	Positive	Positive	Negative	1	1	0	0
03-08-010	36	0	0	1	0	3	1	0	0	0	0	Positive	Positive	Negative	✖	✖	✖	✖
03-08-015	58	0	0	0	0	1	0	0	0	0	0	Positive	Positive	Negative	1	0	0	0
03-08-022	144	0	0	2	1	2	1	2	1	1	1	Positive	Positive	Negative	1	1	1	1
03-08-032	18	0	0	0	0	0	0	3	1	0	0	Positive	Positive	Negative	✖	✖	✖	✖
03-08-036	12	0	0	1	0	0	0	0	0	0	0	Positive	Positive	Negative	1	0	0	0
03-08-043	60	0	0	1	0	0	0	0	0	0	0	Positive	Positive	Negative	1	0	0	0
03-08-047	12	0	0	1	0	0	0	0	0	0	0	Positive	Positive	Positive	1	0	0	0
03-08-049	74	0	0	2	1	0	0	2	1	1	0	Positive	Positive	Negative	1	1	1	1
03-08-072	62	0	0	0	0	2	1	3	1	0	1	Positive	Positive	Negative	1	0	1	0
03-08-075	59	0	0	0	0	0	0	1	0	0	0	Positive	Positive	Positive	1	0	0	0
03-08-077	25	0	0	2	1	2	1	3	1	1	1	Positive	Positive	Negative	1	1	1	1
03-08-078	292	1	0	0	0	3	1	2	1	0	1	Positive	Negative	Negative	1	0	1	0
03-08-085	60	0	0	0	0	0	0	3	1	0	0	Negative	Negative	Positive	1	0	1	0
03-08-109	58	0	0	0	0	0	0	2	1	0	0	Positive	Positive	Positive	1	0	1	0

Supplementary Table 1: Full dataset used in survival analyses (page 5)

Patient ID	Followup (months)	OS	BrCa Events	IGF1R Score	IGF1R bin ‡	pIGF1R Score	pIGF1R bin ‡	SphK Score	SphK bin ‡	IGF1R-SphK Comb ❖	pIGF1R-SphK Comb ❖	ER status	PR status	HER2 status	Anti-estrogen therapy (AET)	AET-IGF1R	AET-SphK	AET-IGF1R-SphK
03-08-125	61	0	0	2	1	1	0	2	1	1	0	Positive	Positive	Negative	1	1	1	1
03-08-126	58	0	0	2	1	0	0	2	1	1	0	Positive	Positive	Positive	1	1	1	1
03-08-130	67	0	0	0	0	0	0	2	1	0	0	Positive	Equivoca	Positive	1	0	1	0
03-09-007	72	0	0	0	0	0	0	2	1	0	0	Negative	Negative	Negative	0	0	0	0
03-09-020	61	0	0	2	1	3	1	3	1	1	1	Positive	Positive	Negative	1	1	1	1
03-09-022	61	0	0	2	1	3	1	3	1	1	1	Positive	Positive	Negative	1	1	1	1
03-09-026	167	0	0	0	0	2	1	1	0	0	0	Positive	Positive	Negative	1	0	0	0
03-09-027	60	0	0	0	0	0	0	3	1	0	0	Negative	Negative	Negative	✖	✖	✖	✖
03-09-029	60	0	0	2	1	0	0	2	1	1	0	Positive	Positive	Positive	1	1	1	1
03-09-032	59	0	0	1	0	0	0	1	0	0	0	Positive	Positive	Positive	1	0	0	0
03-09-034	60	0	0	0	0	2	1	2	1	0	1	Positive	Positive	Negative	1	0	1	0
03-09-035	60	0	0	0	0	0	0	1	0	0	0	Positive	Positive	Positive	1	0	0	0
03-09-041	61	0	0	1	0	0	0	3	1	0	0	Positive	Negative	Negative	1	0	1	0
03-09-077	60	0	0	0	0	1	0	2	1	0	0	Positive	Positive	Negative	1	0	1	0
03-09-117	63	0	0	1	0	0	0	2	1	0	0	Negative	Negative	Negative	✖	✖	✖	✖
03-09-148	60	0	0	2	1	1	0	3	1	1	0	Negative	Negative	Negative	0	0	0	0
04-07-006	61	0	✻	0	0	2	1	2	1	0	1	Negative	Negative	Negative	✖	✖	✖	✖
04-07-012	78	0	0	1	0	3	1	3	1	0	1	Positive	Positive	Negative	1	0	0	0
04-07-016	71	0	0	0	0	3	1	2	1	0	1	Positive	Positive	Negative	1	0	0	0
04-07-046	3	1	0	1	0	0	0	2	1	0	0	Positive	Negative	Positive	0	0	0	0
04-07-060	61	0	1	0	0	1	0	1	0	0	0	Negative	Negative	Negative	1	0	0	0
04-07-065	42	0	0	1	0	1	0	0	0	0	0	Negative	Negative	Positive	0	0	0	0
04-07-071	42	0	0	1	0	0	0	0	0	0	0	Negative	Negative	Positive	0	0	0	0
04-07-072	59	0	0	1	0	0	0	2	1	0	0	Positive	Negative	Positive	1	0	1	0
04-08-002	37	0	0	1	0	0	0	1	0	0	0	Positive	Positive	Positive	1	0	0	0
04-08-025	47	0	0	0	0	0	0	1	0	0	0	Negative	Negative	Positive	0	0	0	0
04-08-057	4	0	0	1	0	0	0	2	1	0	0	Positive	Positive	Positive	✖	✖	✖	✖
04-08-061	61	0	0	2	1	1	0	1	0	0	0	Positive	Positive	Positive	1	1	0	0
04-08-085	30	0	0	2	1	1	0	2	1	1	0	Positive	Positive	Positive	1	1	1	1
04-08-136	29	0	0	0	0	0	0	0	0	0	0	Negative	Negative	Positive	0	0	0	0
04-08-182	22	0	0	1	0	1	0	2	1	0	0	Positive	Negative	Positive	0	0	0	0
04-08-215	30	0	✻	1	0	1	0	0	0	0	0	Positive	Positive	Positive	0	0	0	0
04-09-016	29	0	0	0	0	0	0	1	0	0	0	Positive	Positive	Positive	0	0	0	0
05-07-001	60	0	0	2	1	3	1	2	1	1	1	Negative	Positive	Positive	1	1	1	1
05-07-013	67	1	1	1	0	1	0	0	0	0	0	Positive	Positive	Positive	1	0	0	0
05-07-020	59	0	0	0	0	0	0	0	0	0	0	Negative	Negative	Negative	0	0	0	0
05-07-028	67	0	0	1	0	0	0	1	0	0	0	Negative	Negative	Positive	0	0	0	0
05-07-033	62	0	0	1	0	0	0	2	1	0	0	Negative	Positive	Positive	1	0	1	0
05-07-037	61	0	0	1	0	0	0	1	0	0	0	Positive	Positive	Positive	1	0	0	0
05-07-053	59	0	0	1	0	0	0	1	0	0	0	Positive	Positive	Positive	1	0	0	0
05-07-108	30	0	0	0	0	1	0	2	1	0	0	Negative	Negative	Negative	✖	✖	✖	✖
05-07-130	55	0	0	0	0	1	1	2	1	0	1	Negative	Negative	Positive	0	0	0	0
06-08-004	63	0	0	0	0	0	0	3	1	0	0	Negative	Negative	Negative	0	0	0	0

✻: Alive - disease status unknown

‡: Binary score where 0 or 1 = low (0); 2 or 3 = high(1)

❖: Combined score where none or one high = 0; both high = 1

✖: Data unavailable
