# Supplementary figures and images for: Insulin-like growth factor receptor and sphingosine kinase are prognostic and therapeutic targets in breast cancer
Source: BMC Cancer. 2017 Dec 5;17:820. doi: 10.1186/s12885-017-3809-0 (PMC5718000; doi:10.1186/s12885-017-3809-0)

**
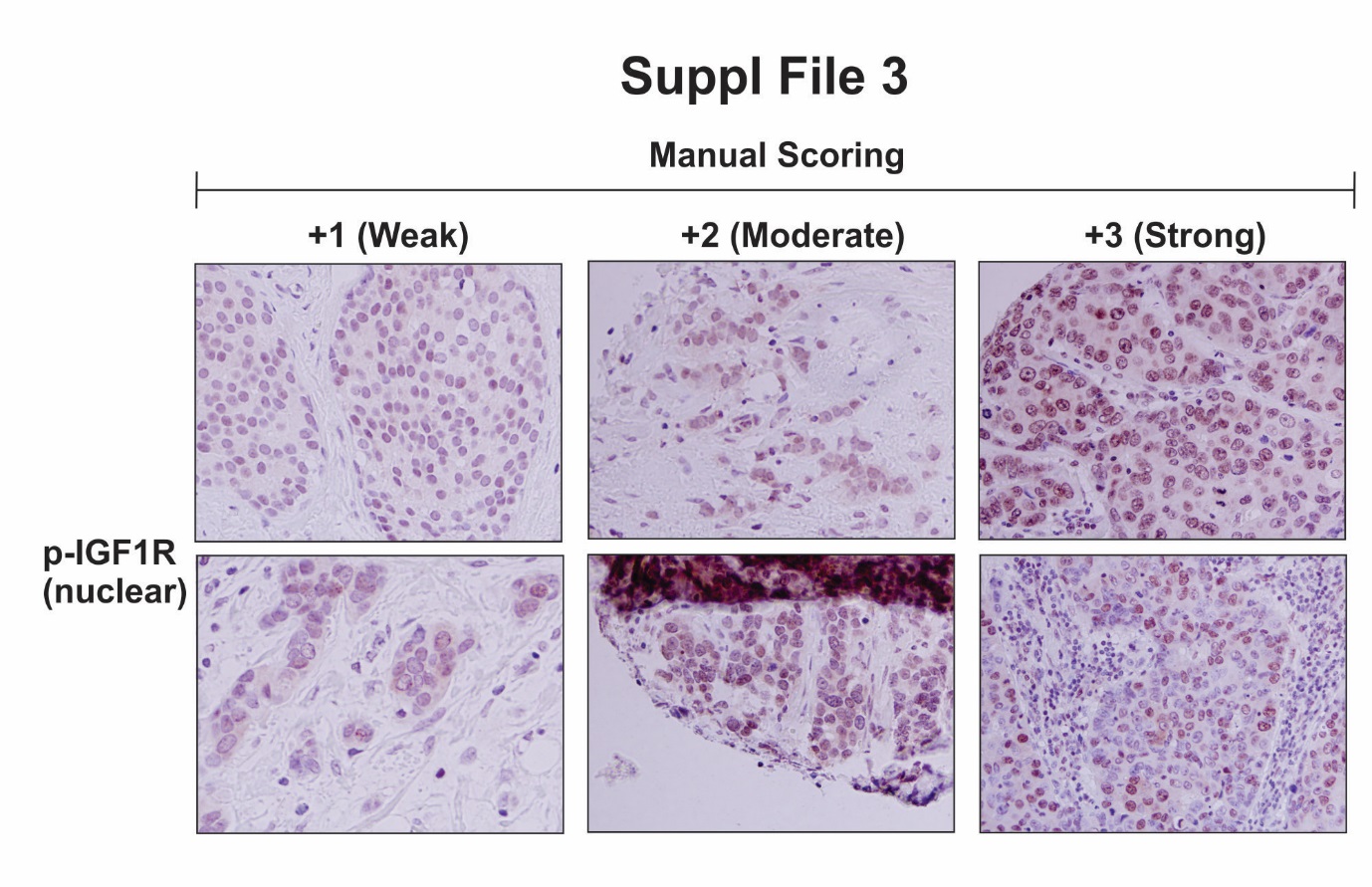
**

Supplement: Supplementary file 2 — Immunohistochemistry and manual scoring analysis of Australian Breast Cancer Tissue Bank patient samples. Refer to Fig. 1 for experimental details. (DOCX 332 kb) [file 12885_2017_3809_MOESM2_ESM.docx]

**
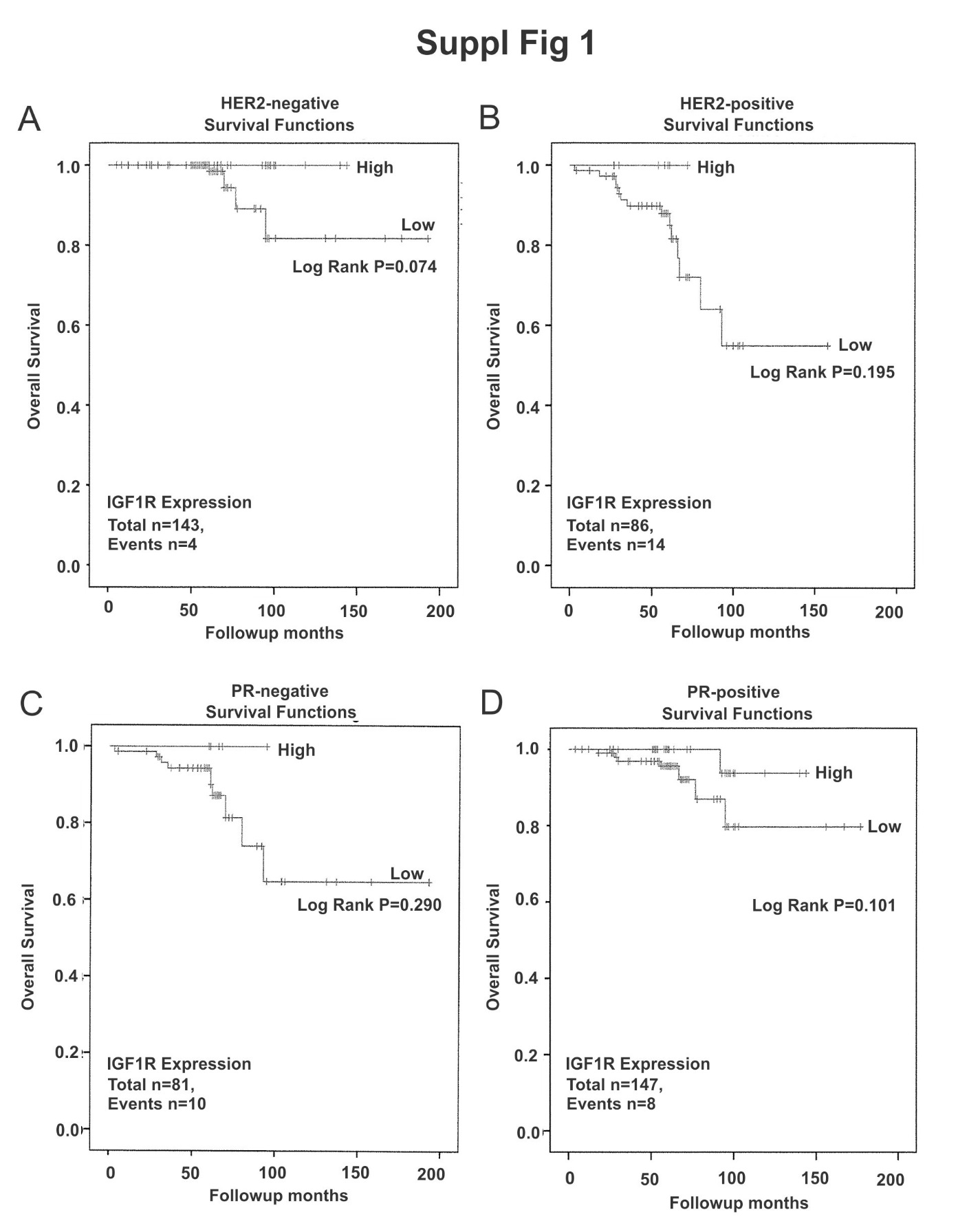
**

Supplement: Supplementary file 3 — Prognostic survival outcomes in relation to IGF1R protein expression in HER2 and PR-positive and negative breast cancer tissues. Kaplan-Meier analysis was performed to measure the overall survival (OS) following stratification for high vs. low IGF1R protein expression, stratified for HER2 and PR expression: A. IGF1R (HER2-negative); B. IGF1R (HER2-positive); C. IGF1R (PR-negative) and D. IGF1R (PR-negative) as described under Methods. (DOCX 264 kb) [file 12885_2017_3809_MOESM3_ESM.docx]

**
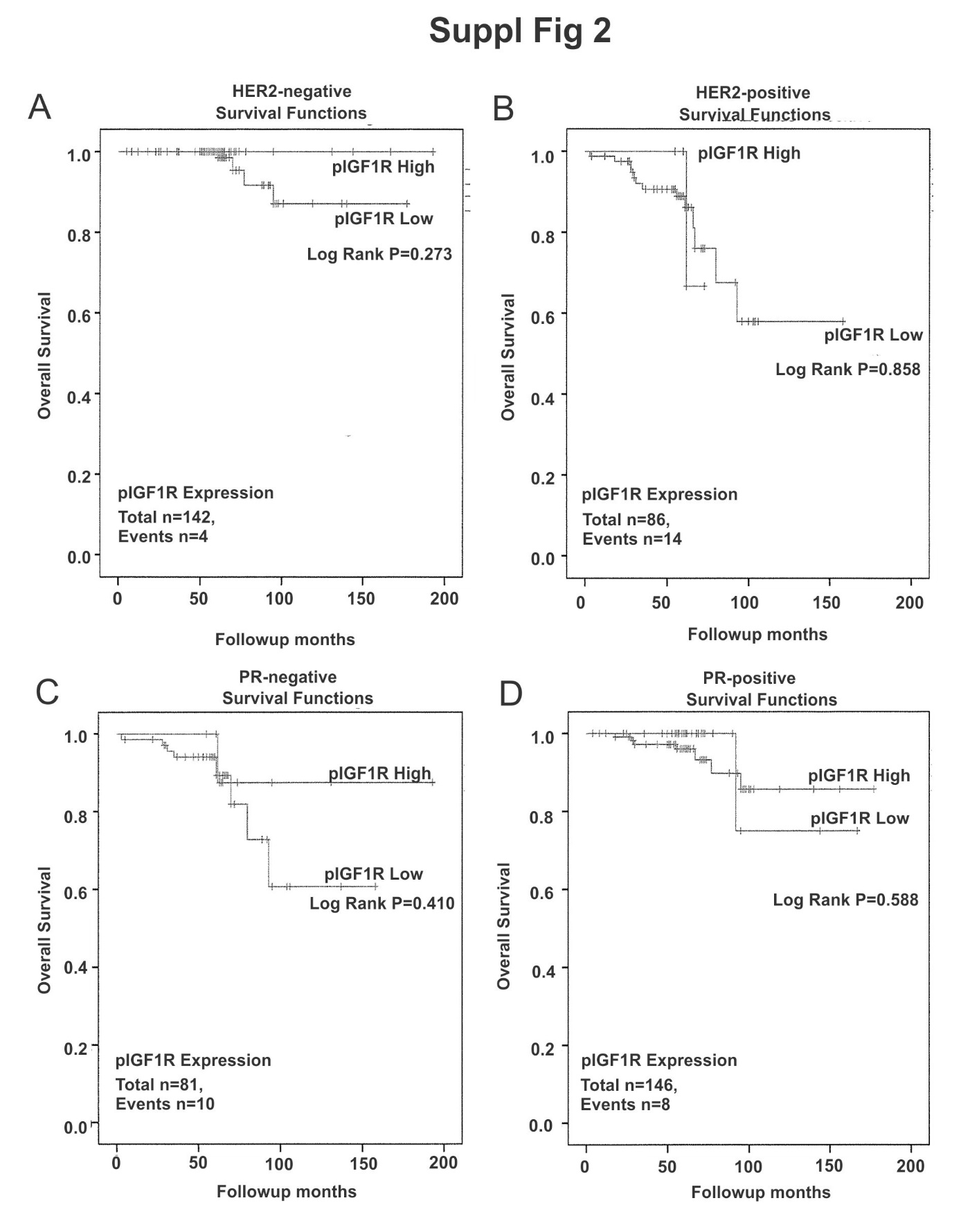
**

Supplement: Supplementary file 4 — Survival outcomes in relation to p-IGF1R protein expression in HER2 and PR-positive and negative breast cancer tissues. Kaplan-Meier analysis was performed to measure the overall survival (OS) following stratification for high vs. low p-IGF1R protein expression, stratified for HER2 and PR expression: A. p-IGF1R (HER2-negative); B. p-IGF1R (HER2-positive); C. p-IGF1R (PR-negative) and D. p-IGF1R (PR-negative) as described under Methods. (DOCX 279 kb) [file 12885_2017_3809_MOESM4_ESM.docx]

**
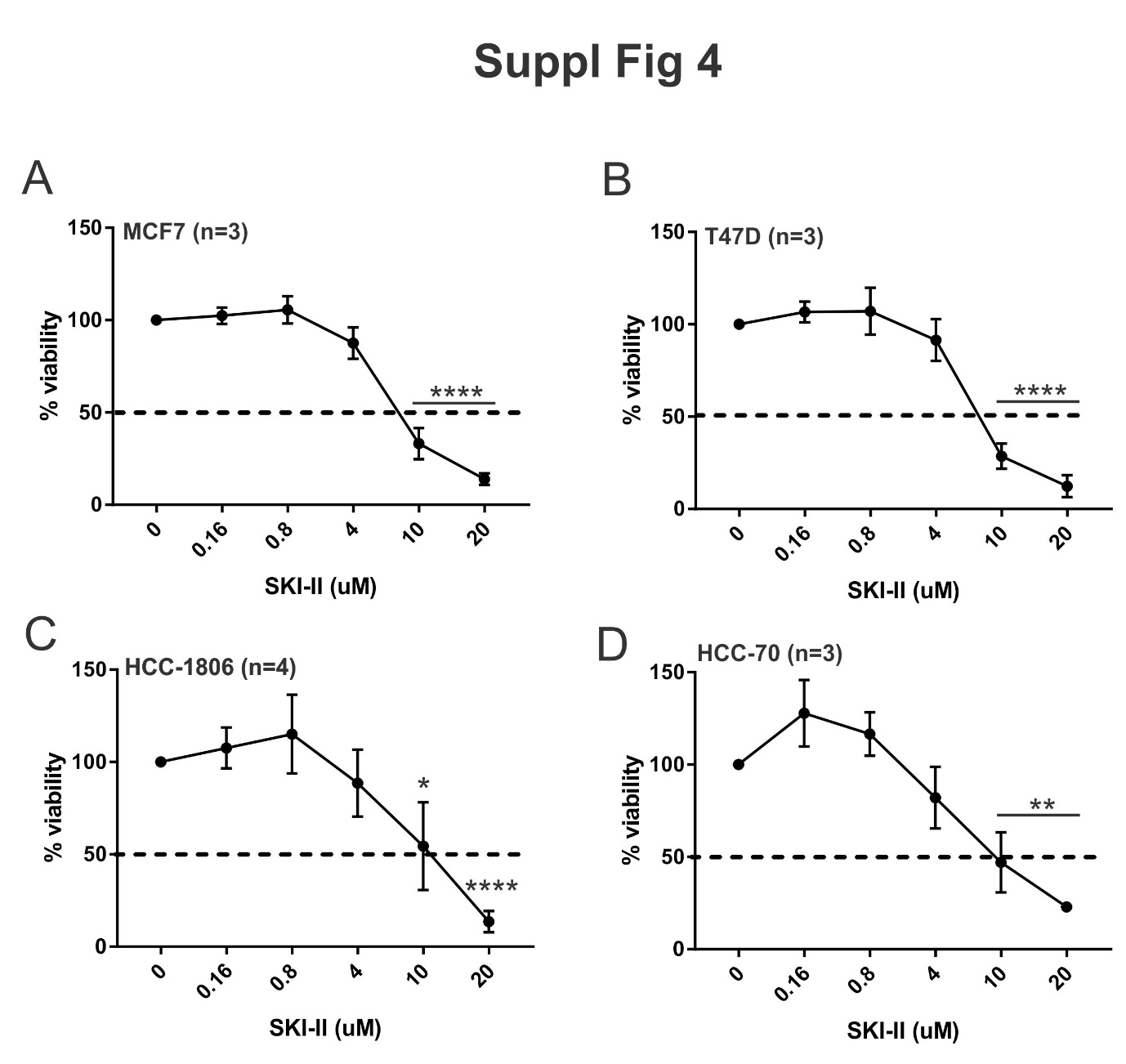
**

Supplement: Supplementary file 5 — Breast cancer cell viability dose curves in response to SKI-II. Refer to Fig. 5 for experimental details. A. MCF7, B. T47D, C. HCC-1806 and D. HCC70 were treated with single agents of the SphK1 inhibitor (SKI-II; 0.16, 0.8, 4, 10 and 20 μM) for 96 h. Graphs depict experimental data normalized to zero treatment vehicle control and 1-way ANOVA followed by Tukey’s test was performed to determine significance between treatment groups and significance accepted p-values *p < 0.05, **p < 0.01, ***p < 0.001 and ****p < 0.0001. (DOCX 186 kb) [file 12885_2017_3809_MOESM5_ESM.docx]

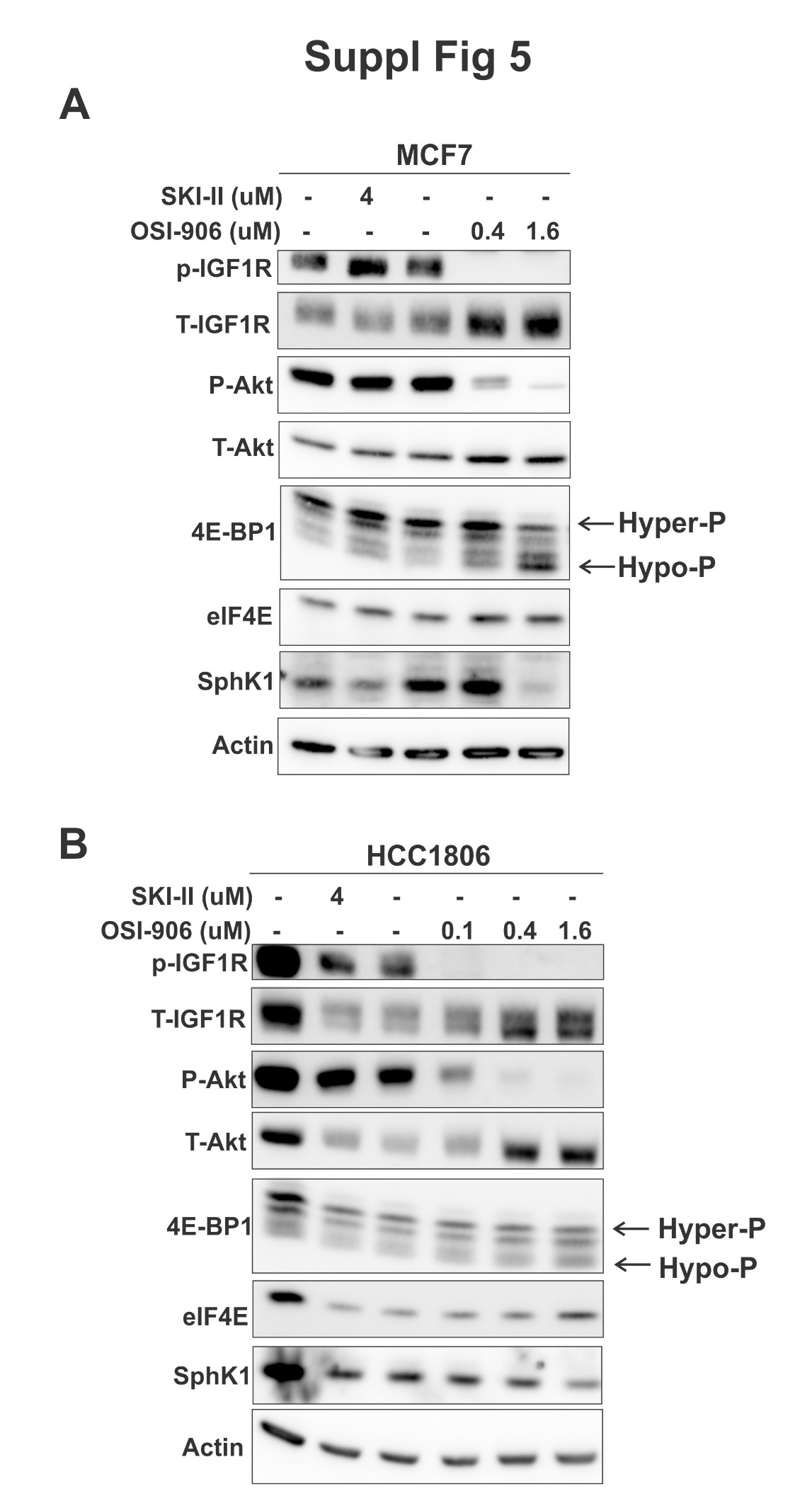

Supplement: Supplementary file 6 — OSI-906 inhibits p-IGF1R and IGF1R signaling factors in breast cancer cell-lines. 3 × 10^5^ ER-positive A. MCF7 and ER-negative B. HCC-1806 breast cancer cells were plated per well of a 6-well plate and cultured for 24 h and subsequently treated with the dual IGF1R/InsR dual tyrosine kinase inhibitor (OSI-906; 0.1, 0.4 and 1.6 μM) and/or SphK1 inhibitor (SKI-II; 4 μM) for 24 h. Protein lysates were collected and 20 μg of protein was used for immunoblot analysis to measure changes to IGF1R signaling (i.e. AKT and eIF4E-BP1 (protein translation)) and SphK1 steady-state protein expression levels. B-actin was used as a loading control. Abbreviations: Hyper-P = hyper-phosphorylation and Hypo-P = hypophosphorylated. (DOCX 255 kb) [file 12885_2017_3809_MOESM6_ESM.docx]

**
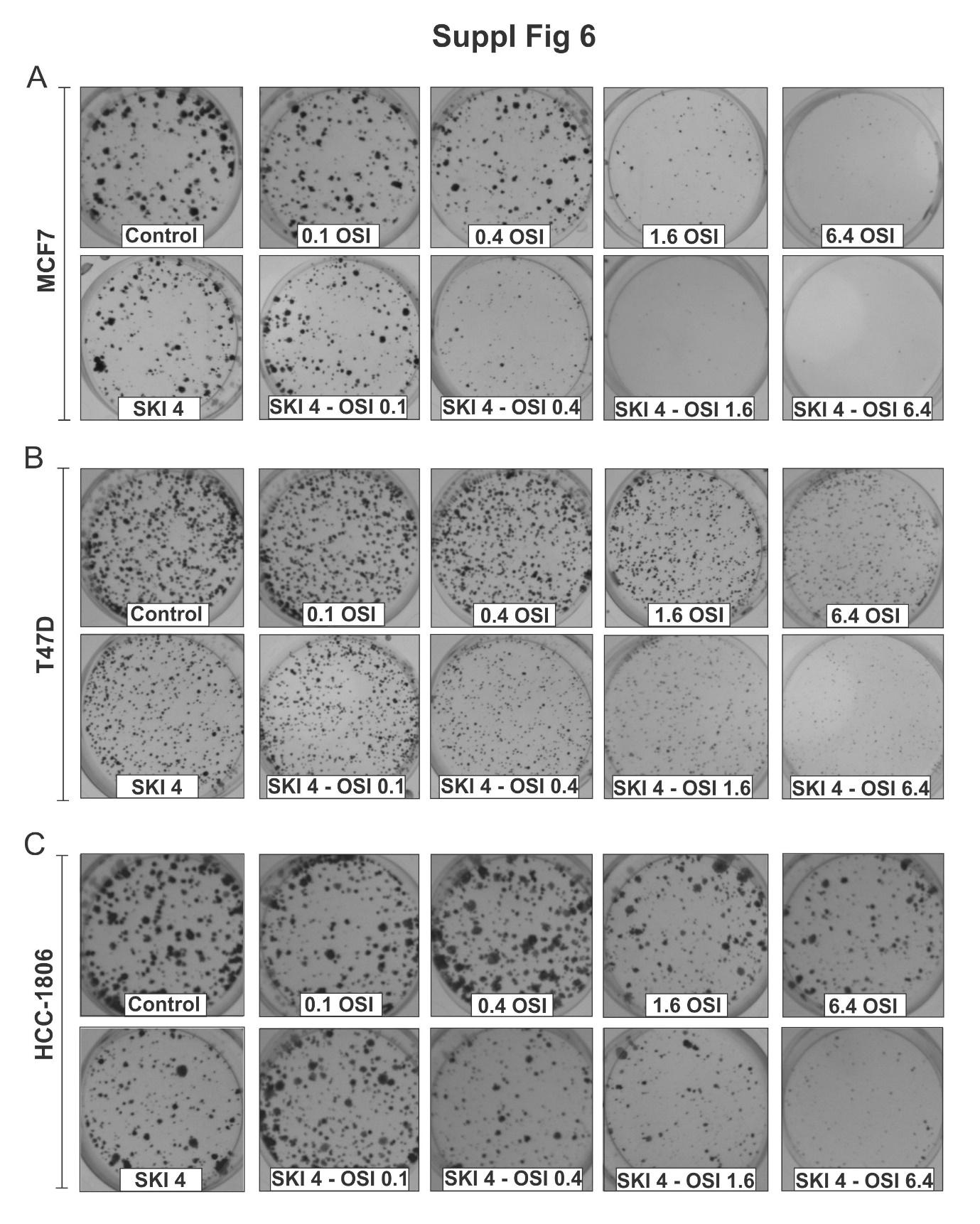
**

Supplement: Supplementary file 7 — Effects of co-targeting IGF1R and SphK1 on colony formation in breast cancer cell-lines. Refer to Fig. 5 for experimental details. Images represent individual wells of colonies following drug treatments for 10-14 d of ER-positive A. MCF7 and B. T47D and ER-negative C. HCC1806 breast cancer cell-lines. Images were captured using FujiFilm Luminescent Image Analyzer LAS-300 (Stamford, CT) and single colonies were counted using open colony forming unit (CFU) software (http://opencfu.sourceforge.net/). (DOCX 545 kb) [file 12885_2017_3809_MOESM7_ESM.docx]
